# Inhibition of the PI3K-AKT-MTORC1 axis reduces the burden of the m.3243A>G mtDNA mutation by promoting mitophagy and improving mitochondrial function

**DOI:** 10.1080/15548627.2024.2437908

**Published:** 2024-12-12

**Authors:** Chih-Yao Chung, Kritarth Singh, Preethi Sheshadri, Gabriel E Valdebenito, Anitta R. Chacko, María Alicia Costa Besada, Xiao Fei Liang, Lida Kabir, Robert D. S. Pitceathly, Gyorgy Szabadkai, Michael R. Duchen

**Affiliations:** a Department of Cell and Developmental Biology and Consortium for Mitochondrial Research, UCL, London, UK; b Cellular and Molecular Neurobiology of Parkinson’s Disease, Research Center for Molecular Medicine and Chronic Diseases (CIMUS), University of Santiago de Compostela, Spain; c Department of Neuromuscular Diseases, UCL Queen Square Institute of Neurology, London, UK; d NHS Highly Specialised Service for Rare Mitochondrial Disorders, Queen Square Centre for Neuromuscular Diseases, The National Hospital for Neurology and Neurosurgery, London, UK; e Department of Biomedical Sciences, University of Padua, Padua, Italy; f The Francis Crick Institute, London, UK

**Keywords:** m.3243A>G, mitochondria, mitophagy, mtDNA mutations, nutrient signaling, PI3K-AKT-MTORC1

## Abstract

Mitochondrial DNA (mtDNA) encodes genes essential for oxidative phosphorylation. The m.3243A>G mutation causes severe disease, including myopathy, lactic acidosis and stroke-like episodes (MELAS) and is the most common pathogenic mtDNA mutation in humans. We have previously shown that the mutation is associated with constitutive activation of the PI3K-AKT-MTORC1 axis. Inhibition of this pathway in patient fibroblasts reduced the mutant load, rescued mitochondrial bioenergetic function and reduced glucose dependence. We have now investigated the mechanisms that select against the mutant mtDNA under these conditions. Basal macroautophagy/autophagy and lysosomal degradation of mitochondria were suppressed in the mutant cells. Pharmacological inhibition of any step of the PI3K-AKT-MTORC1 pathway activated mitophagy and progressively reduced m.3243A>G mutant load over weeks. Inhibition of autophagy with bafilomycin A_1_ or chloroquine prevented the reduction in mutant load, suggesting that mitophagy was necessary to remove the mutant mtDNA. Inhibition of the pathway was associated with metabolic remodeling – mitochondrial membrane potential and respiratory rate improved even before a measurable fall in mutant load and proved crucial for mitophagy. Thus, maladaptive activation of the PI3K-AKT-MTORC1 axis and impaired autophagy play a major role in shaping the presentation and progression of disease caused by the m.3243A>G mutation. Our findings highlight a potential therapeutic target for this otherwise intractable disease.

**Abbreviation**: ΔΨ_m_: mitochondrial membrane potential; 2DG: 2-deoxy-D-glucose; ANOVA: analysis of variance; ARMS-qPCR: amplification-refractory mutation system quantitative polymerase chain reaction; Baf A1: bafilomycin A_1_; BSA: bovine serum albumin; CQ: chloroquine; Cybrid: cytoplasmic hybrid; CYCS: cytochrome c, somatic; DCA: dichloroacetic acid; DMEM: Dulbecco’s modified Eagle’s medium; DMSO: dimethylsulfoxide; EGFP: enhanced green fluorescent protein; LC3B-I: carboxy terminus cleaved microtubule-associated protein 1 light chain 3 beta; LC3B-II: lipidated microtubule-associated protein 1 light chain 3 beta; LY: LY290042; MAP1LC3B/LC3B: microtubule associated protein 1 light chain 3 beta; MELAS: mitochondrial encephalomyopathy, lactic acidosis and stroke-like episodes; MFC: mitochondrial fragmentation count; mt-Keima: mitochondrial-targeted mKeima; mtDNA: mitochondrial DNA/mitochondrial genome; MTOR: mechanistic target of rapamycin kinase; MTORC1: MTOR complex 1; OA: oligomycin+antimycin A; OxPhos: oxidative phosphorylation; DPBS: Dulbecco’s phosphate-buffered saline; PPARGC1A/PGC-1α: PPARG coactivator 1 alpha; PPARGC1B/PGC-1β: PPARG coactivator 1 beta; PI3K: phosphoinositide 3-kinase; PINK1: PTEN induced kinase 1; qPCR: quantitative polymerase chain reaction; RNA-seq: RNA sequencing; RP: rapamycin; SQSTM1/p62: sequestosome 1; TEM: transmission electron microscopy; WT: wild-type

## Introduction

Mitochondria maintain cellular bioenergetic homeostasis by coupling cell metabolism with multiple cell processes, acting as a hub for multiple key cell signaling pathways [1]. Human mitochondrial DNA (mtDNA) encodes 13 peptides as subunits of the electron transport chain complexes and 24 RNAs that execute mitochondrial translation [1]. Pathogenic mutations of mtDNA are a major cause of primary mitochondrial diseases, a group of devastating diseases with varied presentation and severity. The m.3243A>G mutation, one of the most prevalent mtDNA mutations, is a point mutation on position 3243, affecting the *MT-TL1* (mitochondrially encoded tRNA-Leu [UUA/G] 1) gene (the tRNA recognizes the codons UUA/G for leucine). The mutation is estimated to affect 3.7–5.3:100,000 people [2] and is associated with a multisystem disease – most notably with mitochondrial encephalomyopathy, lactic acidosis and stroke-like episodes/MELAS or a less severe syndrome of maternally inherited diabetes and deafness/MIDD in some patients. Most patients show heteroplasmy, carrying both normal and mutated mtDNA, and a higher mutant burden is generally associated with more severe disease. Unfortunately, no consensus approach for treating MELAS syndrome nor disease-modifying treatment is yet available, so the therapeutic approach is limited to symptomatic management [3]. Of note, the estimated MELAS carrier frequency is 236:100,000 [4], while the estimated prevalence is much lower, implying that most carriers are either asymptomatic or exhibit mild symptoms that are not clinically recognized.

Our previous study systematically characterized metabolic remodeling and altered cell signaling in patient-derived fibroblasts and cytoplasmic hybrid/cybrid cells bearing the heteroplasmic m.3243A>G mutation [5]. In brief, we identified constitutive activation of the PI3K-AKT-MTORC1 signaling in the mutant cells, which is strongly associated with an altered metabolite profile, redox imbalance, oxidative stress, and glucose dependence [5]. Remarkably, inhibition of any step of the pathway substantially reduced the mutant load and rescued mitochondrial bioenergetic function cell-autonomously [5]. These findings suggested that pharmacological intervention in this signaling pathway represents a potential therapeutic target for patients with this dreadful disease [5]. However, the downstream mechanisms by which inhibition of the PI3K-AKT-MTORC1 axis select against the mutant mtDNA remains unknown.

Crosstalk between the PI3K-AKT-MTORC1 axis and mitochondrial quality control pathways, including mitophagy, mitochondrial biogenesis and mitochondrial shaping mechanisms, is critically involved in regulating mitochondrial energy homeostasis [6]. The accumulation of damaged mitochondria and defective mitophagy is a hallmark of mtDNA diseases, suggesting that the functional consequences of the pathogenic mutant mtDNA alone are insufficient to activate mitophagy and drive selection against the mutation [7–10]. It is still unclear whether chronic activation of the PI3K-AKT-MTORC1 axis suppresses the mitophagy pathway, permitting the accumulation of dysfunctional mitochondria in cells carrying the m.3243A>G mutation. Therefore, we have now further investigated the mechanisms that reduce the mutant mtDNA burden in this disease.

The present study has revealed that the constitutive activation of the PI3K-AKT-MTORC1 axis in the mutant cells suppressed macroautophagy/autophagy and the lysosomal degradation of mitochondria. Inhibition of this pathway restored autophagic flux and reduced the mutant load through the activation of mitophagy. We also found that short-term inhibition of the PI3K-AKT-MTORC1 pathway promoted recovery of the mitochondrial membrane potential (ΔΨ_m_) and improved oxidative phosphorylation (OxPhos) that preceded the fall in mutant load, suggesting that activation of the pathway plays a role, at least partially, in shaping the metabolic phenotype observed in the m.3243A>G mutant cells beyond the direct bioenergetic effects of the mutation itself. Moreover, the short-term increase in ΔΨ_m_ and OxPhos capacity in response to the drug treatments proved to be important for subsequent effective mitophagy against the mutant mtDNA. Together, effective mitophagy triggered by inhibiting the PI3K-AKT-MTORC1 axis was necessary to eliminate the mutant m.3243A>G mtDNA.

## Results

### Impaired autophagic flux in cells carrying the m.3243A>G mutation is associated with constitutive activation of the PI3K-AKT-MTORC1 axis

In our previous study, RNA-seq data revealed strong enrichment of PI3K-AKT and MTOR pathways in patient-derived fibroblasts carrying the m.3243A>G mtDNA mutation [5], which was confirmed in muscle biopsies by immunofluorescence and re-analysis of published RNA-seq datasets from muscle biopsies of patients with MELAS [11]. These data argue that activation of the PI3K-AKT-MTORC1 axis is a common feature of m.3243A>G mutation both in vivo and in vitro. Analysis of the differentially expressed genes in patient-derived fibroblasts also identified autophagy as an enriched cellular process in patient fibroblasts (*p* = 0.00295). Re-analyzing a published dataset [12] derived from 143B cybrid cells bearing the m.3243A>G mutation using the Generally applicable gene-set (GAGE) method confirmed both upregulated PI3K-AKT and autophagy pathways (Table S1).

**Figure 1.**
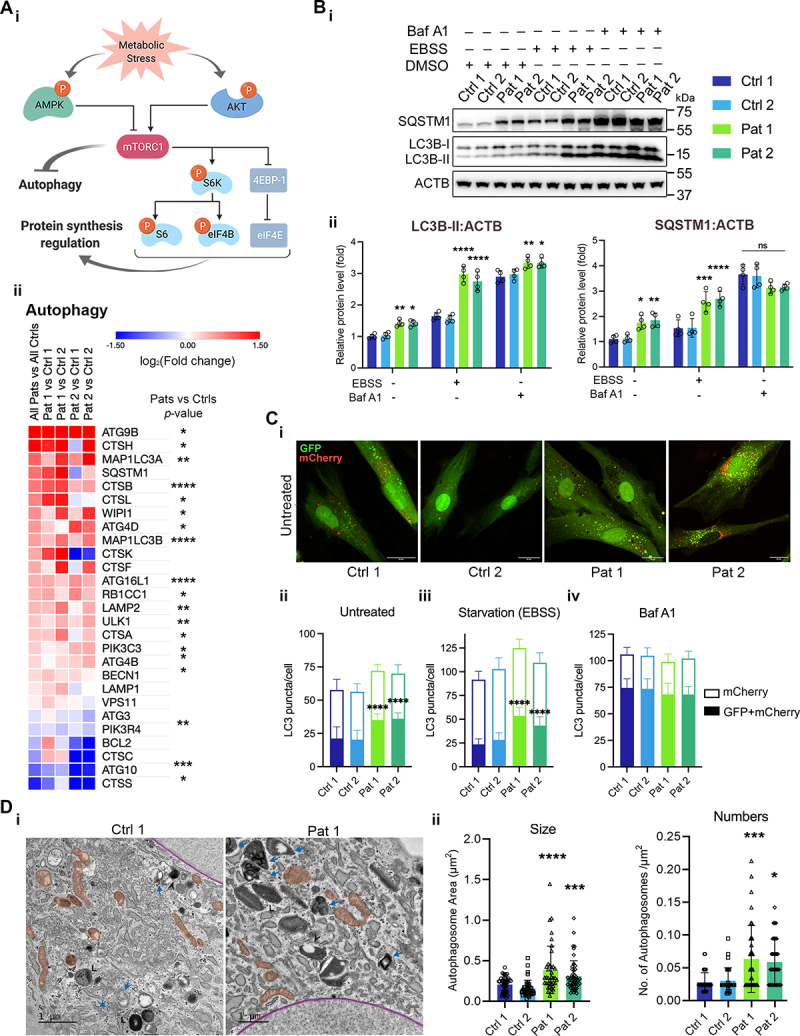
Chronically activated PI3K-AKT-MTORC1 axis in the m.3243A>G mutant cells is associated with impaired autophagy. (A) A scheme showing the regulation of autophagy and protein translation by the PI3K-AKT-MTORC1 pathway (i). Detailed analysis of the mRNA expression of multiple genes involved in the autophagy pathway in patient fibroblasts, showing consistent differences in a wide array of genes involved (ii). (B) Immunoblot of LC3B and SQSTM1 in patient fibroblasts (i) under basal, starvation (EBSS) or bafilomycin A_1_ (Baf A1, 100 nM) conditions (quantified in ii, *n* = 3–4 independent biological samples) also shows an accumulation of LC3B-II in the patient cells without an overall fold increase in autophagic flux that is the conversion of LC3B-I to LC3B-II. (C) Confocal imaging of live cells transfected with the autophagy reporter, mCherry-GFP-LC3B, as a measure of autophagic flux in patient fibroblasts (i and iii, *n* > 100 cells from three independent experiments). The numbers of mCherry and GFP+mCherry puncta under basal (ii), EBSS (iii) and the Baf A1 treatment (iv) were further quantified, showing an increase of GFP+mCherry puncta in patient fibroblasts under untreated and ebss-treated condition. Scale bar: 20 μm. (D) Representative TEM image of control and Pat 1 fibroblasts cytoplasmic area showing autophagosomal structure (blue arrowhead). Black arrowhead shows autolysosomal structures characterized by the presence of electron-dense lysosomal membranes and a large electron-lucent area, indicating degradation of intralumenal material. “L” denotes lysosomes characterized by spherical and electron-dense membrane folds. Mitochondria and nuclear envelope are shown as red and magenta-segmented areas, respectively (i). Scale bar: 1 μm. Quantification of autophagosomal area and number per square micron showed increased size and number in both Pat 1 and Pat 2 fibroblasts (ii). Plots represent the data from three independent experiments with *n* ≥ 30 TEM images. Data in (B-D) are represented as mean ± S.D and were analyzed by one-way ANOVA with Tukey’s multiple comparisons test (*p < 0.05, **p < 0.01, ***p < 0.001, ****p < 0.0001).

We, therefore, explored the status of autophagic flux associated with the activation of the PI3K-AKT-MTORC1 axis in the mutant cells (Figure 1A). RNA expression of genes involved in autophagy was highly upregulated in the patient fibroblasts (Figure 1A). We measured MAP1LC3B/LC3B and SQSTM1/p62 (sequestosome 1) turnover using immunoblotting in cells treated with nutrient-rich or starvation medium with or without the lysosomal inhibitor bafilomycin A_1_ (Baf A1), which inhibits the V-ATPase and thus inhibits lysosomal acidification (Figure 1B). In comparison to controls, mutant cells showed a significant conversion of LC3B-I to the lipidated form (LC3B-II) and SQSTM1 accumulation both under normal and starvation conditions. Inhibition of autophagic flux by Baf A1 led to further accumulation of LC3B-II and SQSTM1 in both control and mutant cells; however, the non-significant difference in the level of SQSTM1 (Figure 1B) and LC3B-II (Fig. S1A) between Baf A1 treated control and mutant cells suggested that the accumulation of LC3B-II in mutant cells resulted from reduced autophagosome-lysosome fusion and/or lysosomal dysfunction rather than an increased autophagic flux. We also measured autophagic flux using a mCherry-EGFP-LC3B tandem reporter. The EGFP is quenched in an acidic environment following the fusion of autophagosomes with lysosomes, so that autolysosomes are seen as red puncta (Figures 1C and S1Bi). Consistent with the above result, control cells showed an increased number of red-LC3B puncta, representing the formation of mature autolysosomes under both basal and starvation conditions. In contrast, the number of double-positive puncta, showing a complete merge of GFP and mCherry signal, were significantly increased in mutant cells under basal and starvation conditions (Figures 1Cii-iii, S1Bii-iii and S1Ci). This effect was observed in both control and mutant cells after Baf A1 treatment, confirming the accumulation of autophagosomes and inhibition of autophagic flux in mutant cells (Figures 1Civ and S1Cii).

We next used transmission electron microscopy (TEM) imaging to identify autophagosomal structures and to measure the size and number based on the characteristic double-limiting membrane vesicle containing electron-dense cytoplasmic materials or organelles at various stages of degradation (Figure 1D). TEM analysis revealed a significant increase in the mean area of autophagosomes and in the number of autophagosomes per square micron in cells bearing the m.3243A>G mutation, suggesting impaired maturation of autophagosomes to autolysosomes (Figure 1D).

To determine whether impaired autophagy pathway is a common feature of cells carrying mtDNA mutations, we also examined the autophagic flux by LC3B-II immunoblotting in cells carrying a different heteroplasmic mtDNA point mutation – the m.8993T>G – a kind gift from the Minczuk lab [13] – and found no significant difference among cells bearing 0%, 50% or 80% mutant loads (Fig. S1D). Our previous study has also shown that there was no difference in the phosphorylation of AKT or RPS6 in these cells carrying the m.8993T>G mutation [5]. Altogether, these data confirm that autophagic flux was impaired in cells carrying the m.3243A>G mutation and may be caused by the activation of the PI3K-AKT-MTORC1 axis (Table S1) in the mutant cells, but also suggest that these pathways may be mutation specific and are not a generalized feature of cells carrying mutations of mtDNA, at least within the scope of this work.

### Endocytic uptake and lysosomal degradation are altered in cells with m.3243A>G mutation

**Figure 2.**
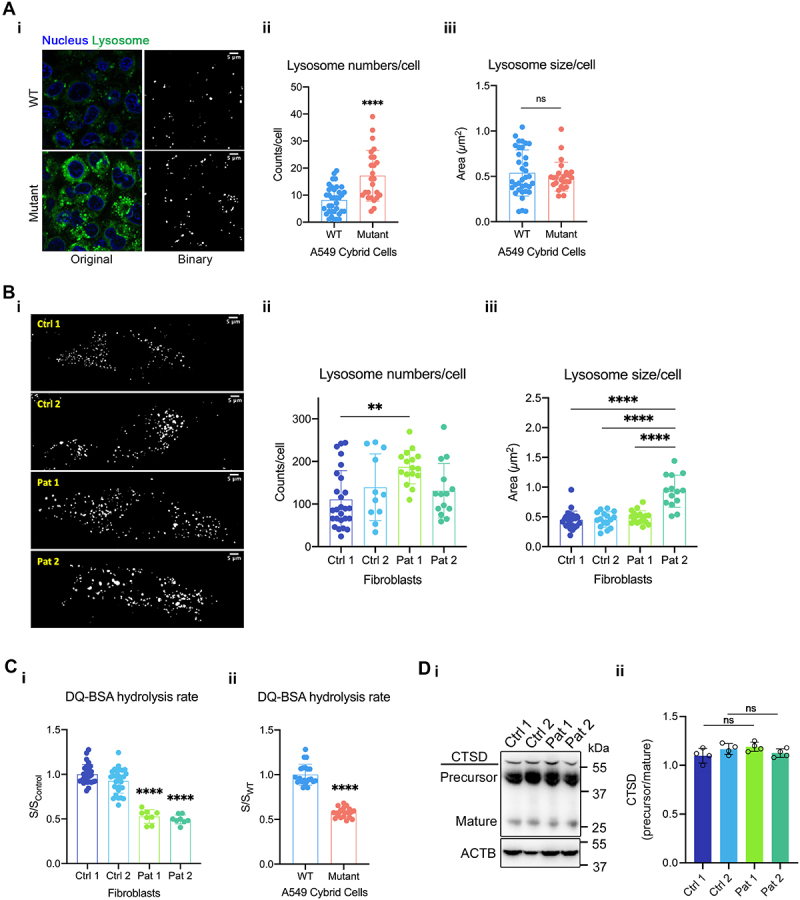
Lysosomal form and function are altered in cells carrying the m.3243A>G mutation. (A) Representative images of WT and mutant cybrid cells labelled for nucleus with Hoechst 33,342 and lysosomes with LysoTracker Green (i; scale bar: 5 μm). Binary images, as shown in (i), were used to quantify particle number and area for the measurement of mean lysosome numbers/cell (ii) and size/cell (iii). The plot represents the data from *n* = 50 cells from three independent experiments. (B) Binarized images of LysoTracker Green-labelled control and patient fibroblasts in (i) were measured to quantify lysosome numbers/cell (ii) and size/cell (iii). The plot represents the data from *n* = 50 cells from three independent experiments. Scale bar: 5 μm. (C) Lysosomal proteolytic activity was measured by DQ-BSA assay and showed that the activity was reduced both in the patient fibroblasts (i) and in the A549 cybrid cells (ii), compared to controls. The plot represents the data from three independent experiments. (D) CTSD immunoblot from cell lysates of control and patient fibroblasts showing precursor and mature forms (i). The ratio of CTSD’s precursor to mature form was measured from two independent experiments, and no significant change was observed (ii). Data are represented as mean ± S.D. and were analyzed by one-way ANOVA with Tukey’s multiple comparisons test for fibroblasts and by unpaired t test for cybrid cells (*p < 0.05, **p < 0.01, ***p < 0.001, ****p < 0.0001).

While the fusion of autophagosomes with lysosomes is essential for the clearance of autophagosomes, the acidic lumenal environment and active hydrolases of autolysosomes are also crucial for autophagic degradation. Accumulation of autophagosomes as measured by autophagic flux assays and TEM analysis suggested a possible alteration of lysosomal function in cells carrying the m.3243A>G mutation. This prompted us to investigate lysosomal morphology and function in the mutant cells. The average number and size of lysosomes per cell were measured using LysoTracker Green, which labels acidic organelles (Figure 2A–B). The average number, but not size, of lysosomes was significantly increased in the A549 cybrid cells carrying the m.3243A>G mutation compared to the WT controls (Figure 2A). Similarly, the average number of lysosomes was significantly higher in Pat 1 fibroblasts (with a mutant load of 86.2 ± 2.3%) than in Ctrl 1 (Figure 2B), while the average size of lysosomes in Pat 2 fibroblasts (with a mutant load of 30.3 ± 3.5%) was significantly higher than in the controls (Figure 2B).

While the increase in lysosomal number in the cybrid cells and Pat 1 fibroblasts might be associated with lysosomal stress, observation of enlarged lysosomes in Pat 2 fibroblasts suggested a possible disruption of lysosomal function. To test this, we measured lysosomal proteolytic activity using DQ-BSA, a self-quenched albumin probe which is taken up by endocytosis and generates fluorescence upon degradation within lysosomes. Cells were loaded with DQ-BSA, and the increase in fluorescence was monitored for 4 hours to determine the rate of DQ-BSA degradation. Interestingly, both the mutant cybrid cells and patient fibroblasts showed a diminished rate of DQ-BSA degradation, which suggested that lysosomal proteolysis is suppressed in mutant cells (Figure 2C). Decrease in lysosomal proteolysis could result from a change in the abundance or activity of lysosomal proteases. Cathepsins are lysosomal proteases which become proteolytically active at low pH in lysosomes. Therefore, we examined the precursor and the mature form of one of the major cathepsins, CTSD/cathepsin D, by immunoblotting (Figure 2D). The ratio of precursor/mature form of CTSD in patient fibroblasts was not significantly changed, showing a normal CTSD activation (Figure 2D). These data suggested that impaired autophagic flux and autophagosome accumulation may indirectly affect lysosomal function, leading to abnormal lysosomal morphology and proteolytic activity in cells with the m.3243A>G mutation.

### Mitophagy is inhibited in cells carrying the m.3243A>G mutation

**Figure 3.**
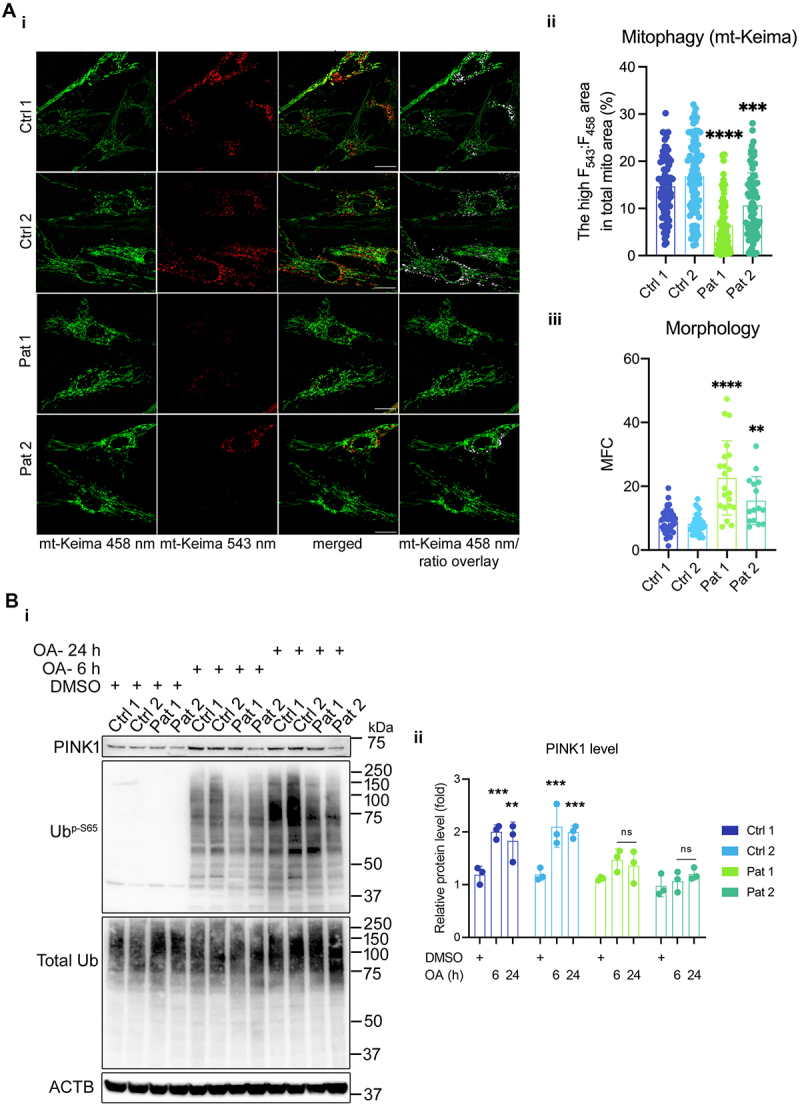
Mitophagy is impaired in cells carrying the m.3243A>G mutation. (A) Live cell imaging of mt-Keima expressing control and patient fibroblasts showing mitophagy status at 543 nm excitation. The overlay of mt-Keima emission at 458 nm and the ratio image shows the relative level of mitophagy events in control vs patient fibroblasts. The individual mitophagy events, as represented in ratio image (i), were quantified and plotted as the percentage of high F_543_:F_458_ ratio area to the total mitochondria area (ii), showing a significant reduction in “mitophagosome” maturation in patient fibroblasts (*n* ≥ 80–100 cells from six independent experiments; scale bar: 20 μm). Mitochondrial morphology was also quantified using the mitochondrial fragmentation count (MFC), confirming fragmented mitochondrial networks in the patient fibroblasts exhibited (iii). (B) Cell lysates from DMSO and oligomycin (1 μg/ml) and antimycin a (1 μM) treated control and patient fibroblasts were immunoblotted for PINK1, total ubiquitin and phospho-ubiquitin (Ser65) to assess PINK1 activation (i). PINK1 immunoblot from three independent experiments was analyzed to quantify the changes in its levels over time after drug treatment (ii). Data are represented as mean ± S.D. and were analyzed by one-way ANOVA with Tukey’s multiple comparisons test for fibroblasts (*p < 0.05, **p < 0.01, ***p < 0.001, ****p < 0.0001).

Given the impaired autophagic flux and abnormal lysosomal activity observed in m.3243A>G mutant cells, we sought to examine the mitophagy pathway of mitochondrial quality control – selective autophagic removal of mitochondria. To visualize the delivery of impaired mitochondria to lysosomes, we transfected the fibroblasts with mitochondrial-targeted mKeima/mt-Keima [14], which undergoes a shift in excitation spectrum within acidic lysosomes, indicating the conversion of autophagosome to autolysosomes. Mitochondrial morphology was also quantified using the mitochondrial fragmentation count (MFC). A ratio of signal excited at 543 nm and 458 nm (F_543_:F_458_) provides a quantitative readout of mitophagy activity (Figure 3A). The control fibroblasts exhibited punctate structures showing a strong mt-Keima signal at 543 nm, which indicated that a subpopulation of mitochondria was delivered to lysosomes. Quantifying the ratio image also demonstrated a high ratio F_543_:F_458_ signal corresponding to the increased formation of autolysosomes containing degraded mitochondria. In contrast, the patient fibroblasts exhibited fragmented mitochondrial networks (higher MFC), displaying a strong fluorescence at 458 nm but a diminished mt-Keima signal at 543 nm (Figure 3A). The subpopulation of mitochondria with a high ratio F_543_:F_458_ signal was significantly decreased in the mutant cells, consistent with a defect in autolysosome maturation in the patient fibroblasts. Next, we used COX8-EGFP-mCherry reporter (EGFP-mCherry targeted to the mitochondrial matrix, similar to mCherry-EGFP-LC3B) [15] as a mitophagy reporter in the cybrid cells. The proportion of mitophagy-positive cells was quantified by flow cytometry in which a threshold of red fluorescence (P2 gate) was used to select the mitophagy population (Fig. S1E). The flow cytometry data revealed an increase in the P2 population in response to mitochondrial depolarization induced by oligomycin and antimycin A (OA) in control cells, and the population was suppressed when the control cells were co-treated with Baf A1. However, in the mutant cybrid cells, the mitophagy population did not alter significantly when cells were treated with either OA or OA+Baf A1, suggesting an accumulation of autophagosomes containing mitochondria or their impaired maturation.

Mitochondrial fission and lowered ΔΨ_m_ are important for PINK1-PRKN/Parkin-dependent mitophagy [16,17]. Our previous study reported that patient fibroblasts bearing the heteroplasmic m.3243A>G mutation showed fragmented mitochondria with a reduced ΔΨ_m_ [5], which might be expected to increase the probability of dysfunctional mitochondria being targeted for mitophagy. Therefore, we measured PINK1 accumulation and activation by measuring ubiquitin phosphorylation at Ser65 following treatment with OA at different time points (Figure 3B). The control fibroblasts showed a time-dependent increase in PINK1 activation as indicated by the Ub p-S65 blot and increased PINK1 levels in response to OA-induced mitophagy. In comparison, we did not observe a significant increase in PINK1 activity or its levels in patient fibroblasts (Figure 3B) or in the cybrid cells (Fig. S1F), indicating that PINK1-dependent mitophagy initiation in response to mitochondrial depolarization is altered or suppressed in cells carrying the m.3243A>G mutation. Altogether, these findings suggest that constitutive activation of the PI3K-AKT-MTORC1 signaling suppresses mitophagy, possibly permitting the accumulation of dysfunctional mitochondria in the m.3243A>G mutant cells.

### Inhibition of the PI3K-AKT-MTORC1 axis reduces m.3243A>G mutant load by increasing autophagic flux and activating mitophagy

Our previous study demonstrated that the long-term treatment (over ~6 weeks) of the mutant cells with inhibitors of the PI3K-AKT-MTORC1 axis, including rapamycin (RP) and LY294002 (LY), reduced mutant load and reversed the biochemical/bioenergetic consequences of the mutation cell-autonomously [5]. We next asked what mechanisms select against the mutant mtDNA following the drug treatments.

It seemed plausible that removing dysfunctional mitochondria via mitophagy may reduce the mutant load in cells with the mtDNA mutation [18,19]. Indeed, long-term treatment with LY or RP significantly increased the accumulation of LC3B-II (autophagosome) in the presence of the lysosome inhibitors chloroquine (CQ, which inhibits lysosomal function by alkalinizing the lumen) compared to untreated cells (Fig. S2A-B). Measurement of autophagic flux using the mCherry-GFP-LC3B probe showed a significant increase in the formation of mature autolysosomes (red puncta) as early as 24 h after drug treatment and during long-term treatment in the cybrid cells and patient fibroblasts (Fig. S2C-D). The ratio of green:red puncta numbers decreased significantly following drug treatments, suggesting increased autophagic flux in the m.3243A>G mutant cells. Analysis of the cybrid cells transfected with mCherry-GFP-LC3B by flow cytometry also showed an increase in cells with low GFP intensity after RP or LY treatment (Fig. S2E). Similar results were also found in the cybrid cells and the patient fibroblasts treated with another class 1 PI3K inhibitor, GDC0941 (GDC; 1 μM), and an inhibitor of AKT, MK2206 (MK; 1 μM) (Fig. S2F).

Additionally, measurements of lysosomal proteolysis using DQ-BSA showed that lysosomal degradation in mutant cells significantly recovered after 24-h drug treatments (Fig. S3A). To determine whether increased autophagic flux and improved lysosomal degradation affected LC3B recruitment to mitochondria in mutant cells following the PI3K-AKT-MTORC1 inhibition, we immunolabelled LC3B and CYCS (cytochrome c, somatic) in cybrid cells for different durations and measured the colocalization between LC3B and CYCS signal. The image analysis showed a significant increase in co-localization between LC3B and CYCS over the whole period of drug treatments, suggesting an upregulation of LC3B recruitment to dysfunctional mitochondria and activation of mitophagy (Fig. S3B).

**Figure 4.**
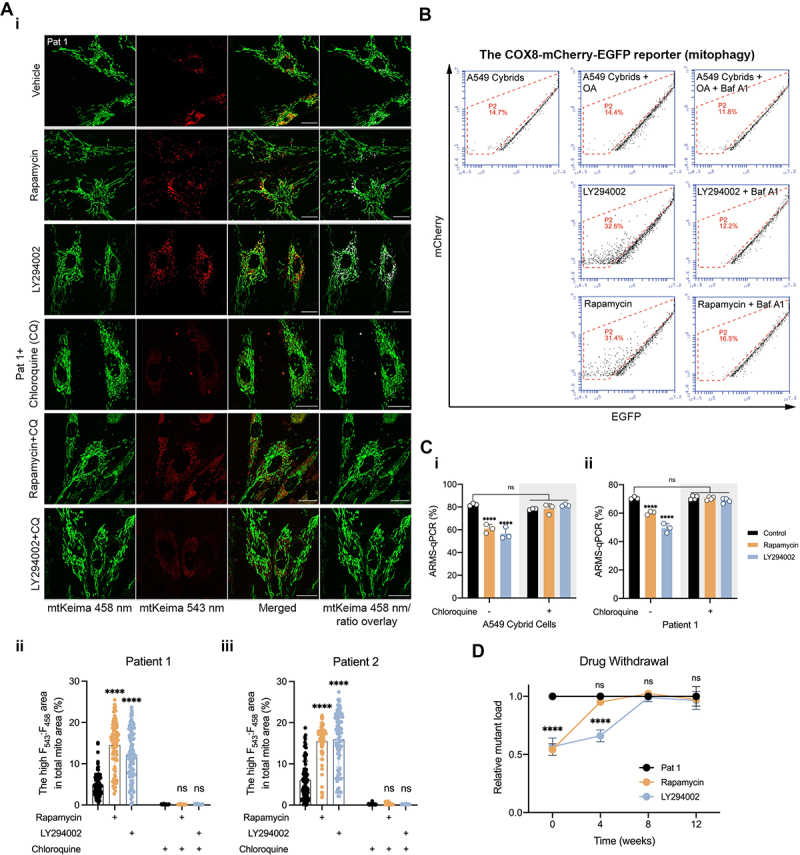
Autophagy activation following the inhibition of the PI3K-AKT-MTORC1 axis promotes mitophagy and reduces the m.3243A>G mutant load. (A) The mitophagy reporter (i), mt-Keima, was used to quantify mitophagy in patient fibroblasts treated with LY or RP. The mitochondrial area fraction engulfed in autolysosomes in both patient 1 (ii) and patient 2 (iii) were quantified and showed a significant increase in mitophagy in both patient cell lines when treated with RP ad LY (*n* = 82–95 cells), which was completely prevented by CQ treatment (*n* = 32–57 cells from five independent experiments; scale bar: 20 μm). (B) Analysis of A549 cybrid cells transfected with COX8-egfp-mCherry under various conditions (*n* = 3 independent biological samples for each condition) using flow cytometry showed an increase in the proportion of mitophagy-positive cells in response to LY, RP and OA-induced mitophagy while co-treatment with Baf A1 reversed the effect. (C) A549 cybrid cells (i) and Pat 1 fibroblasts (ii) were cultured with the inhibitors, RP and LY, as in all prior experiments but in combination with CQ (10 μM), showing that CQ completely prevented the decrease of mutant load in response to inhibition of the PI3K-AKT-MTORC1 pathway (*n* = 3–4 independent experiments). (D) Following the withdrawal of the drugs from Pat 1 fibroblasts previously treated with RP or LY (for 12 weeks), the mutant load of the treated cells reversed back to the level comparable to the untreated cells after 8 weeks. Data in (A and C-D) are represented as mean ± S.D. and were analyzed by one/two-way ANOVA with Tukey’s multiple comparisons test (*p < 0.05, **p < 0.01, ***p < 0.001, ****p < 0.0001).

To quantify whether the increase in LC3B recruitment to mitochondria also improves its lysosomal degradation, we transfected the patient fibroblasts with the mt-Keima probe and measured the F_543_:F_458_ ratio. After 24 h of drug treatments (Figure 4A), the F_543_:F_458_ mt-Keima ratio increased significantly in patient fibroblasts with a significant increase in red puncta (engulfed mitochondria). Similarly, the cybrid cells were transfected with COX8-mCherry-EGFP. The long-term drug treatment with RP and LY dramatically increased the proportion of cells with low GFP intensity, suggesting the restoration of mitophagy (Figure 4B). These findings confirm that inhibition of the PI3K-AKT-MTORC1 pathway restores autophagic flux and promotes mitophagy in the m.3243A>G mutant cells.

Our previous work has shown that inhibiting the PI3K-AKT-MTORC1 pathway led to a progressive decrease in mtDNA mutant load in both the Pat 1 fibroblasts and the cybrid cells, while the Pat 2 fibroblasts showed a progressive increase in mutant load over time in culture, which was prevented by the inhibitors. The progressive decrease in mutant load and increase in autophagic flux following the PI3K-AKT-MTORC1 inhibition in the m.3243A>G mutant cells strengthened the idea that upregulated mitophagy drives selection against the mutant mtDNA and decreased the mutant load. To explore this further, we quantified mitophagy in patient fibroblasts expressing mt-Keima treated with RP or LY, in the presence of CQ to inhibit lysosomal function. The cotreatment with CQ prevented the effect of LY and RP on mitophagy, decreasing the high ratio F_543_:F_458_ signal to levels comparable to untreated patient fibroblasts (Figure 4A). Remarkably, CQ treatment over 6 weeks in the Pat 1 fibroblasts and cybrid cells completely prevented the decrease of mutant load in response to inhibition of the PI3K-AKT-MTORC1 axis (Figure 4C), demonstrating that increased mitophagy is necessary to reduce the burden of mutant mtDNA. Moreover, after drug withdrawal, the mutant load gradually reversed back to the level comparable to the untreated cells after 8 weeks in Pat 1 fibroblasts previously treated with RP- and LY (Figure 4D). Together, these data show that effective autophagy triggered by inhibiting the PI3K-AKT-MTORC1 axis is necessary to eliminate the m.3243A>G mtDNA.

The change in mutant load could also result from the elimination of mutant mtDNA by reducing the total mtDNA copy number and then amplifying the dominant mtDNA. We therefore quantified the total mtDNA copy number of the drug-treated cells at a series of time points but found no significant changes between the drug-treated and untreated mutant cells (Fig. S4A); that is, the total mtDNA copy number remained stable across the duration of drug treatments. The expression of TFAM and PPARGC1A/PGC-1α and PPARGC1B/PGC-1β – master genes that regulate mitochondrial biogenesis – were also measured. Surprisingly, the protein levels of TFAM by immunoblotting were downregulated in the mutant cells after 4-week drug treatments (Fig. S4B). Similarly, reverse transcription quantitative polymerase chain reaction/RT-qPCR revealed that PPARGC1A- and PPARGC1B-related genes were downregulated after long-term drug treatments (Fig. S4C-D). However, these genes were upregulated 4 days post-treatment in the cybrid cells (Fig. S4Ci). These data indicated that the mtDNA copy number remained the same, and the PPARGC1A-PPARGC1B pathway was not upregulated despite the activation of mitophagy following inhibition of PI3K-AKT-MTORC1 axis in cells carrying the m.3243A>G mutation.

### Promoting OxPhos by pharmacological inhibition of the PI3K-AKT-MTORC1 signaling is required for the reduction of the mutant load in cells carrying the m.3243A>G mutation

**Figure 5.**
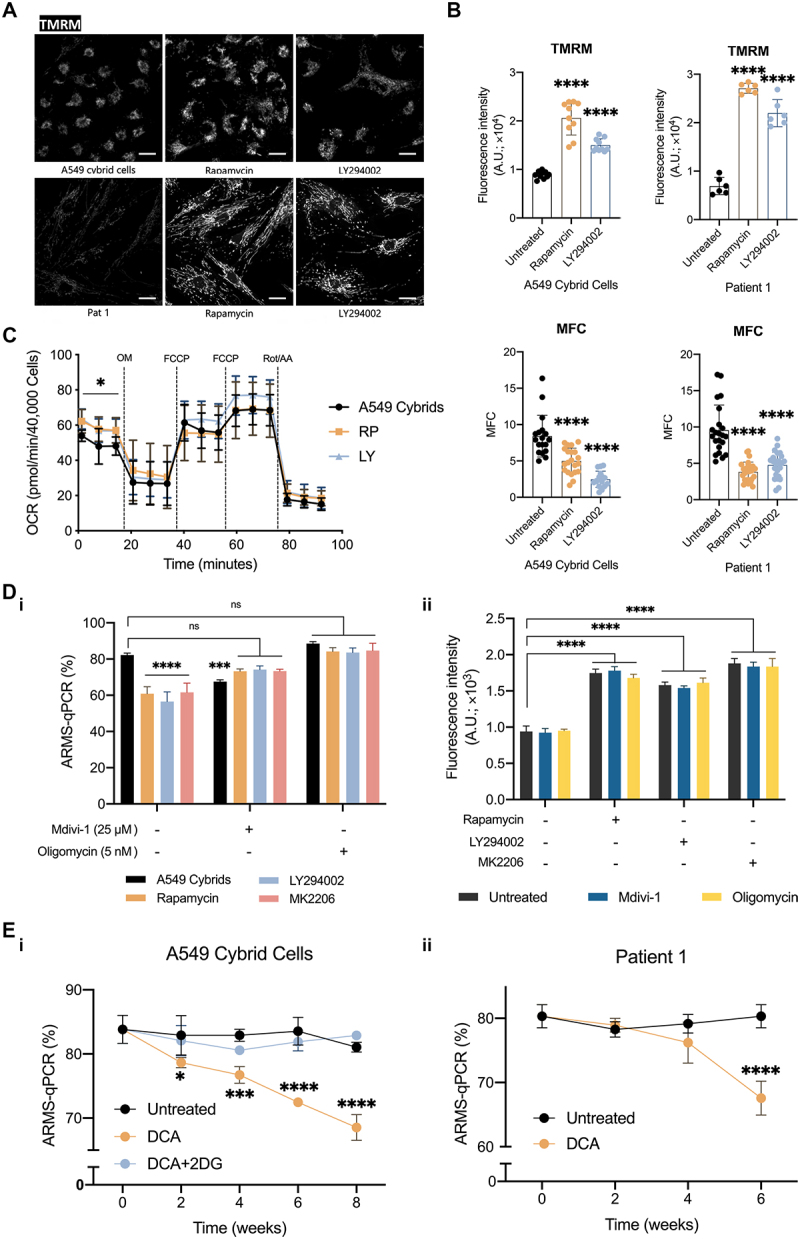
The OxPhos function and mitochondrial fission are required to reduce mtDNA mutant load following inhibition of the PI3K-AKT-MTORC1 axis. (A-B) Representative images (A) showing short-term effects of inhibiting the PI3K-AKT-MTORC1 axis on ΔΨ_m_ (measured using TMRM 25 nM; scale bar: 20 μm) of cells carrying the m.3243A>G mutation. Quantification of the data is shown in (B). The ΔΨ_m_ (the upper panel) of the A549 cybrid cells (*n* = 10; the left panel) and Pat 1 fibroblasts (*n* = 5–6; the right panel) was significantly increased following treatment with RP or LY for 24 h. The MFC (the lower panel) confirmed that mitochondria became less fragmented after drug treatments. (C) Cell respiratory capacity of the A549 cybrid cells treated with LY or RP for 24 h was measured using the Seahorse XFe96 extracellular flux analyzer, showing a significant increase in basal oxygen consumption rate (OCR) after the short-term treatment (*n* = 15). (D) The A549 cybrid cells were cultured with the inhibitors RP and LY as in prior experiments. However, in combination with Mdivi-1 (25 μM) or oligomycin (5 nM), showing that Mdivi-1 or oligomycin prevented the decrease of mutant load in response to the inhibition of the PI3K-AKT-MTORC1 pathway (i; *n* = 3). The ΔΨ_m_ of A549 cybrid cells significantly increased after exposure to RP, LY or MK for 24 h. (E) Sustained treatment of cells over 6 weeks with DCA caused a progressive decrease in relative mutant mtDNA load in the A549 cybrid cells (i) and Pat 1 fibroblasts (ii), while cotreatment with 2DG reversed the effect of DCA on the mutant load in the cybrid cells. Data in (B-E) are represented as mean ± S.D. and were analyzed by one/two-way ANOVA with Tukey’s multiple comparisons test (*p < 0.05, **p < 0.01, ***p < 0.001, ****p < 0.0001).

Removal of mutant mtDNA following inhibition of the PI3K-AKT-MTORC1 axis might also involve metabolic remodeling, allowing mitophagy to remove mutant mtDNA selectively. To answer this, the mitochondrial function of the mutant cells was measured after only 24 h treatment with the inhibitors, long before any significant change in mutant load could be detected. The ΔΨ_m_ was significantly increased in all cell lines – the A549 cybrid cells and Pat 1 fibroblasts – following exposure to RP or LY compared to untreated cells (Figure 5A–B). Mitochondrial morphology was also quantified using the MFC and showed considerable improvement of the mitochondrial network in the mutant cells treated with RP or LY (Figure 5B). Mitochondrial respiration of the RP- or LY-treated A549 cybrid cell also showed a small but significant increase in basal respiration at this early time point (Figure 5C). These data suggest that the bioenergetic defect in the m.3243A>G mutant cells is not only caused directly by the mutation but is, at least, partly secondary to the impact of the altered cell signaling pathways on metabolic pathways. In other words, the rescue in mitochondrial bioenergetics is mediated by a direct effect of inhibiting the PI3K-AKT-MTORC1 signaling.

We further investigated the mechanisms that allow mitophagy to remove dysfunctional mitochondria potentially carrying mutant mtDNA selectively. As previously shown, changes in mitochondrial morphology were observed in cells carrying the m.3243A>G mutation and after drug treatments (Figure 5B). To directly test whether the mitochondrial fragmentation observed in the mutant cells is necessary for mitophagy against mutant mtDNA, the mutant A549 cybrid cells were treated with Mdivi-1 (25 μM), which targets DRP1 and thus inhibits mitochondrial fission [20]. Although we recognize that Mdivi-1 may have off-target effects [20], treatment with Mdivi-1 itself slightly reduced mutant load, but cotreatments of Mdivi-1 with the inhibitors of the PI3K-AKT-MTORC1 axis, RP, LY or MK, prevented the reduction of mutant load by the inhibitors (Figure 5Di), confirming the importance of fission in response to these treatments.

To determine whether a reduction in ΔΨ_m_ marks mutant mitochondria for mitophagy, the effect of further dissipating the ΔΨ_m_ in mutant mitochondria was tested [10,21]. The cybrid cells were treated with oligomycin (5 nM), which blocks proton flux through the F_1_F_o_-ATP synthase and thus may increase ΔΨ_m_ [21]. As expected, the treatments with the inhibitors of the PI3K-AKT-MTORC1 axis, RP, LY, or MK, increased ΔΨ_m_. In contrast, oligomycin did not act as expected and had no noticeable effect on ΔΨ_m_, either by itself or as cotreatment with RP, LY or MK (Figure 5Dii). Nonetheless, oligomycin is also an inhibitor of ATP synthesis. The treatment slightly increased the mutant load in the A549 cybrid cells and prevented the effect of RP, LY or MK on the reduction of mutant load. Interestingly, PINK1 activity, inferred from phospho-ubiquitin levels in the drug-treated mutant cells, showed no significant difference between the drug treatments and their controls, suggesting that activated mitophagy after drug treatments in the mutant cells was PINK1-PRKN-independent (Fig. S5Ai-iii).

Conversely, we also tested the effect of dichloroacetic acid (DCA, an inhibitor of pyruvate dehydrogenase kinase), which promotes OxPhos through the activation of PDH (pyruvate dehydrogenase), on the mutant load in the A549 cybrid cells and Pat 1 fibroblasts. Treatment with DCA (1 mM over 6 weeks) significantly reduced the mutant load in the cybrid cells (Figure 5Ei). The effect was reversed when the cybrid cells were co-treated with 2-deoxy-D-glucose (2DG, 10 mM), which blocks the glycolytic generation of pyruvate and thus reverses the effect of DCA on OxPhos. Similarly, a significant decrease in the mutant load was also observed in Pat 1 fibroblasts (Figure 5Eii). Altogether, these data support our hypothesis that OxPhos function is critical in the activation of mitophagy and reduction of mutant load in cells with cells carrying the m.3243A>G mutation.

## Discussion

Several studies have observed increased bulk autophagy in a variety of mitochondrial disease models, including cell models bearing mtDNA mutations [7,10,22], the “Deletor” mouse model [23] and muscle biopsies from patients with m.8344A>G mutations [24]. It is plausible that autophagy is upregulated in response to mitochondrial stress [8,25]. Moreover, a previous study [10] has shown that rapamycin induces mitophagy in cells carrying a variety of mtDNA mutations, although the consequent change in mutant load is unknown. The mechanisms by which dysfunctional mitochondria escape from mitochondrial quality control pathways are still unclear. Hypotheses have been proposed, including the “leaky-link” model, which describes how mitochondrial quality control processes might fail and permit the accumulation of mutant mtDNA [26]. We have addressed this fundamental question by pharmacological inhibition of the PI3K-AKT-MTORC1 pathway, which reduces the m.3243A>G mutation load by activating mitophagy and improving OxPhos function.

The PI3K-AKT-MTORC1 pathway regulates autophagy, preventing autophagosome formation and lysosomal biogenesis. In our published RNA-seq [5] and a published microarray dataset [12], in addition to the PI3K-AKT-MTORC1 pathway, autophagy-related genes were also enriched in the m.3243A>G mutant cells (Figure 1A and Table S1). Interestingly, lysosomal dysfunction is also observed in cells with mitochondrial defects, which seem to be independent of decreased ATP availability [8,27]. Our biochemical and imaging studies for autophagic flux and lysosomal function (Figures 1–2 and S1) agree with these studies, showing accumulation of autophagosomes and lysosomal dysfunction in cells carrying the m.3243A>G mutation. A recent study reported that MTORC1 activation may directly suppress lysosomal proteolytic activity by controlling V-ATPase-mediated acidification of lysosomes [28]. Thus, chronic activation of MTORC1 and impaired autophagy observed in the m.3243A>G mutant cells may secondarily impact lysosomal function. In contrast to the m.3243A>G mtDNA mutation, the m.8993T>G mutation had no impact on basal autophagic flux (Fig. S1D). This again suggests that the changes in signaling pathways are mutation specific.

Dysfunctional mitochondria are segregated during fission and subsequently inhibited from fusion into the healthy mitochondrial network, thus targeting these damaged mitochondria for degradation [29,30]. However, mitophagy was not upregulated in patient fibroblasts bearing the heteroplasmic m.3243A>G with a similar phenotype – fragmented mitochondria and low ΔΨ_m_. Another study using induced pluripotent stem cells carrying the m.3243A>G mutation revealed that mitophagy is scarce under basal conditions [22]. This may explain, at least partially, why these deleterious mtDNA mutations escape removal by mitochondrial quality control surveillance mechanisms. Most notably, pharmacological inhibition of PI3K, AKT, or MTORC1 in the patient-derived cells promoted autophagic flux, partially recovered lysosomal proteolysis and activated mitophagy. We further found that mitophagy was absolutely required to reduce the mutant load by these drug treatments (Figures 3 and S3–4). Further research is needed to elucidate the underlying molecular mechanisms driving the interactions among mitochondrial dysfunction, the PI3K-AKT-MTORC1 axis, lysosome functions and autophagy in cells bearing the m.3243A>G mutation.

On the other hand, it may also be possible to eliminate the mutant mtDNA through “the bottleneck” effect [31]. Since autophagy is upregulated and apparently removes mutant mtDNA in response to the drug treatments, we expected to see either a decrease in mtDNA copy number or activation of mitochondrial biogenesis. However, in these cell models treated with inhibitors of the PI3K-AKT-MTORC1 axis, despite the activation of mitophagy, the mtDNA copy number remained the same, and the PPARGC1A-PPARGC1B pathway was not activated over the long-term treatments (Fig. S2). In a recent study, treatment of cells with 2DG was shown to selectively inhibit mutant mtDNA replication, while the treatment did not alter mtDNA copy number [32]. Therefore, further investigations are needed into the mechanisms selecting against the mutant mtDNA and maintaining mtDNA homeostasis in these cell models.

Interestingly, we found that pharmacological inhibition of the PI3K-AKT-MTORC1 axis also had acute effects on mitochondrial bioenergetics, improving the mitochondrial network, ΔΨ_m_ and the OxPhos activity (Figure 4). It is reported that the PI3K-AKT-MTORC1 pathway promotes mitochondrial activity and biogenesis [33,34]. However, several studies have shown that MTORC1 inhibition by rapamycin reduces ROS production and improves mitochondrial bioenergetics in the context of mitochondrial dysfunction [35,36]. These data suggest that the metabolic remodeling in the m.3243A>G mutant cells is not simply caused by the direct consequences of the mutation and is, at least, partly secondary to the altered cell signaling pathways.

It is known that mitochondrial fission supports mitophagy [16,37], while the modest loss of ΔΨ_m_ is insufficient to promote mitophagy [10,38]. It seems clear that the presence of pathogenic mtDNA itself is not sufficient to drive selection against the mutation through mitophagy [5,10]. Therefore, we asked whether mitochondrial fission and loss of ΔΨ_m_ mark mutant mitochondria for selection, presumably by mitophagy, in the drug-treated cells. Thus, Mdivi-1 and oligomycin were used to inhibit mitochondrial fission and manipulate ΔΨ_m_ and OxPhos activity, respectively. Although a recent study has challenged the action of Mdivi-1 as a selective inhibitor of DNM1L/DRP1 [39], the evidence in the literature still favors the initially described bioactivity of Mdivi-1 [20]. We found that Mdivi-1 reversed the effect of the inhibitors of the PI3K-AKT-MTORC1 axis on mutant load, which implies that the action of mitophagy in reducing the mutant burden is PINK1-PRKN dependent since DRP1 ablation reduces the PINK1-PRKN dependent mitophagy [38]. However, the treatment with Mdivi-1 itself also slightly reduced mutant load, similar to the findings of Lin et al [40]. By contrast, other studies demonstrated that inhibition of fission favors propagation of mutant mtDNA [37], while inhibition of fusion promotes the elimination of mutant mtDNA [19]. The Mdivi-1 solo treatment, which had little effect on ΔΨ_m_, suggests that mitochondrial dynamics mediated by DRP1 limit the enrichment of mutant mtDNA in individual organelles. Meanwhile, oligomycin did not act as expected and had no noticeable effect on ΔΨ_m_ in the cybrid cell model. Nonetheless, this could be a net result of increasing ΔΨ_m_ in ATP-generating mitochondria (operating ATP synthase function) and decreasing ΔΨ_m_ in ATP-consuming mitochondria (maintaining ΔΨ_m_ through the reversal of the ATPase). Moreover, the treatment with oligomycin also inhibits ATP synthesis, which complicates the data interpretation.

We hypothesized that inhibition of the PI3K-AKT-MTORC1 axis improves OxPhos, reducing cell dependence on glycolysis and forcing cells to rely on OxPhos for their energy demand, thus allowing healthy mitochondria to operate at their full capacity; consequently, in the mitochondrial quality control process, incompetent mitochondria potentially carrying the mutant mtDNA are segregated by mitochondrial fission, and these mutant mitochondria are recognized and removed by mitophagy. Our experiments showed that activation of OxPhos by DCA reduced the mutant load in the m.3243A>G mutant cells, while treatment with DCA + 2DG in the mutant cybrid cells blocking OxPhos was sufficient to reverse the selective removal of mutant mtDNA. Of note, DCA, due to its ability to reduce lactate production, once entered clinical trials for treating mitochondrial diseases, especially in MELAS patients, but the trials were terminated because of unexpected peripheral nerve toxicity [41]. A previous study also suggested that abolishing OxPhos, not loss of ΔΨ_m_ nor elevating ROS production, is necessary for mitophagy, regardless of the stimulating pathways [42]. Our findings shown in Figure 5 matched the observations of previous studies and the predictions of our hypothesis.

Overall, the data presented here support the “leaky-link” model, describing how mitochondrial quality control processes might fail and permit the accumulation of mutant mtDNA [26]. The model suggests that despite activation of compensatory mechanisms for the mitochondrial dysfunction, the presence of mutant and WT mtDNA gene products in the same mitochondrion could allow intramitochondrial functional complementation (a very leaky link) and thus prevent elimination of mutant mtDNA by mitophagy. Meanwhile, in a less “leaky link”, with closer genotype-phenotype coupling (linked), fission can generate fragmented mitochondria with a low ΔΨ_m_, unable to fuse, which are available for elimination by mitophagy. In our cell models, this is achieved by inhibiting the PI3K-AKT-MTORC1 axis or activating OxPhos activity.

In summary, our study showed that inhibition of the constitutive activation of the PI3K-AKT-MTORC1 axis in the m.3243A>G mutant cells reduced the mutant load in a mitophagy-dependent way. Moreover, short-term inhibition of the PI3K-AKT-MTORC1 axis promoted recovery of the mitochondrial membrane potential and improved oxidative phosphorylation that preceded a fall in mutant load in the m.3243A>G mutant cells, which were critical for mitophagy against the mutant mtDNA. Our findings point to a potential therapeutic target for mitochondrial diseases.

## Materials and methods

### Data and code availability

Our previously published raw and processed RNA-seq data of patient fibroblasts [5] are accessible at the Gene Expression Omnibus under accession GSE175477. The published microarray dataset of osteosarcoma 143B cybrid cells was downloaded from Gene Expression Omnibus with the accession GSE129091 [12].

### Cell lines

The MRC Centre for Neuromuscular Disorders Biobank in London provided control and patient fibroblasts harboring the m.3243A>G mtDNA mutation. At the time of the biopsies, the patient fibroblasts were recovered from two female subjects (a mother and a daughter), who were 59 and 35 years old, respectively. Two controls were matched for age and gender. Diabetes, myoclonus, sensorineural hearing loss, memory loss, myopathy, pigmentary retinopathy, and bipolar affective disorder were the clinical symptoms for patient 1. Diabetes, sensorineural deafness, cerebellar ataxia, myopathy, epilepsy, depression, and cognitive impairment were the symptoms for patient 2. The patient 1 derived line (Pat 1) showed a mutant load of 86.2 ± 2.3%; the patient 2 derived cell line (Pat 2) showed a mutant load of 30.3 ± 3.5% [5]. Ian Holt (Biodondostia Research Institute, San Sebastián, Spain) graciously shared the A549 cybrid cell line containing the m.3243A>G mtDNA mutation, which has a mutant load of 79.0 ± 0.3% [5]. The 143B cybrid cell lines bearing ~ 50% and ~ 80% of the m.8993T>G mtDNA mutant loads were made and shared by the Minczuk lab (MRC Mitochondrial Biology Unit, Cambridge, UK). These cell lines were grown in Dulbecco’s modified Eagle’s medium (Gibco 10566016) with 10% fetal bovine serum and 1% antibiotic-antimycotic (Gibco 15240096) supplements and incubated at 37°C with 5% CO_2_.

### Cell culture and drug treatments

A549 cybrid cells (WT and mutant) and 143B cybrid cells were passaged every 3–4 days at 80% confluence, using 0.25% trypsin-EDTA (Gibco 25200056). Similarly, passages of patient and control fibroblasts were performed in the same manner every week. Media with or without drugs were changed every 2–3 days. Control and patient fibroblasts were routinely checked for mycoplasma using MycoAlert™ Mycoplasma Detection Kit (Lonza, LT07–118). For drug treatments, rapamycin (Cayman Chemical 13346), LY294002 (Cayman Chemical 70920), GDC0941 (Cayman Chemical 11600) and MK2206 (Cayman Chemical 11593) were dissolved in DMSO (Sigma-Aldrich, D2650) at 10 mM for stocks. To assess autophagy, chloroquine (Cayman Chemical 14194) was dissolved in Dulbecco’s phosphate-buffered saline (DPBS; Gibco 14190144) at 50 mM, while bafilomycin A_1_ (Cayman Chemical 11038) was dissolved in DMSO for stock at 200 μM. Oligomycin (Cayman Chemical 11341) was in DMSO at 25 mM, Mdivi-1 (Sigma-Aldrich, M0199) was in DMSO at 50 mM and dichloroacetate (Sigma-Aldrich 347795) was in DPBS at 300 mM. Drugs were subsequently diluted to their working concentrations in media (rapamycin and LY294002, 5 µM; GDC0941 and MK2206, 1 µM; chloroquine, 50 µM; bafilomycin A_1_, 100 nM; oligomycin, 25 nM; Mdivi-1, 25 µM; DCA, 1 mM). The mutant load of A549 cybrid cells, 143B cybrid cells and the fibroblasts of patient 1 without treatments did not change significantly throughout the study as assessed by amplification-refractory mutation system-quantitative polymerase chain reaction (ARMS-qPCR).

### Measurements for mtDNA mutations

Levels of mtDNA mutation were measured using quantitative PCR (qPCR) based on ARMS [43]. DNA was extracted from frozen cells using the DNeasy Blood & Tissue Kit (Qiagen 69506). After measuring DNA samples using NanoDrop, samples were then diluted to 0.4 ng/µl. Master mixes for mutant and wild-type genes were made with ARMS primer working solutions (5 μM, 1 μl each) and SYBR Green JumpStart Taq ReadyMix (Sigma-Aldrich, S4438) together. DNA samples (3 μl) and master mixes (7 μl) were pipetted into a 96-well PCR plate (Bio-Rad, MLL9651) and the CFX96 Touch Real-Time PCR Detection System (Bio-Rad) was used to carry out PCR amplification. Each sample has three technical replicates. Mutant heteroplasmy level (%) was calculated using 1/[1 + (1/2) ^ΔCT^] × 100%, where ΔC_T_ (cycle threshold) = CT_wild-type_ – CT_mutant_. All primer pairs used can be found in Table S2.

### Analysis of published RNA-sequencing and microarray datasets

The autophagy-related gene expression heatmap was generated from the RNA-sequencing data (GSE175477) of patient fibroblasts using Morpheus (https://software.broadinstitute.org/morpheus). The published microarray dataset (GSE129091) was analyzed at a threshold of FDR < 0.05 and a fold change ≥ 1. Kyoto Encyclopedia of Genes and Genomes/KEGG pathway enrichment tree and tables were generated using iDEP 9.1 (http://bioinformatics.sdstate.edu/idep/).

#### SDS-PAGE and immunoblotting

Cells were plated in 60 mm plates for the A549 cybrid cells and 10 cm plates for fibroblasts. To assess autophagic flux, cells were either starved in Earle’s Balanced Salt Solution (EBSS; Gibco 24010043) for 8 h or were treated with 50 μM chloroquine (5 h) or 100 nM bafilomycin A_1_ (24 h). Also, to induce mitophagy, cells were treated with 1 μg/ml oligomycin and 1 μM antimycin A for the indicated time. Cells were then washed with ice-cold DPBS once and lysed using 150–300 µl RIPA buffer (Sigma-Aldrich, R0278) with one cOmplete™ Protease Inhibitor Cocktail (Roche 4693116001) tablet and one PhosSTOP Phosphatase Inhibitor Cocktail (Roche 4906837001) tablet. Cells were then scraped and centrifuged at 16,000 g at 4°C for 30 min. The Pierce BCA Assay Kit (Thermo Scientific 23227) was used to determine protein concentrations in the supernatant. For immunoblotting, 30 µg of protein samples in NuPAGE 4× LDS Sample Buffer (Invitrogen, NP0007) and 2% β-mercaptoethanol (Sigma-Aldrich 63689) were boiled at 99°C for 5 min. Proteins were separated on 4–12% NuPAGE Bis-Tris polyacrylamide gels (Invitrogen, NP0335) or 12% Bis-Tris gels (Invitrogen, NW00122) immersed in MOPS running buffer (Invitrogen, NP0001) and transferred onto PVDF membranes (Millipore, IPFL00010). Membranes were then incubated in Intercept (TBS) Blocking Buffer (Li-COR Biosciences, 927-60001) for 1 h at room temperature. After the addition of primary antibodies diluted in the blocking buffer with 0.1% Tween-20 (Sigma-Aldrich, P1379), membranes were incubated overnight at 4°C on a shaker. Subsequently, membranes were incubated with appropriate secondary antibodies (Li-COR Biosciences; 1:10000; IRDye® 680RD Goat anti-Mouse IgG, 926-68070; IRDye® 800CW Goat anti-Rabbit IgG, 926-32211) for 1 h at room temperature before signals were developed with the LiCOR Odyssey CLx system. Following is the antibodies and dilutions for immunoblotting: anti-LC3B (1:3000; Cell Signaling Technology, 3868), anti-SQSTM1/p62 (1:1000; Abcam, ab56416), anti-PINK1 (1:1000; Abcam, ab23707), anti-CTSD/cathepsin D (1:1000; Cell Signaling Technology, 69854S), anti-TFAM (1:3000; Cell Signaling Technology, 7495), anti-ubiquitin (1:3000; Santa Cruz Biotechnology, sc-166553), anti-phospho-ubiquitin (Ser65; 1:5000; Merck, ABS1513-I) and anti-ACTB/β-actin (1:10000; Santa Cruz Biotechnology, sc-47,778).

#### Measurement of autophagy and mitophagy using mCherry-EGFP-LC3B, mt-Keima or COX8-EGPF-mCherry reporters, respectively

The autophagy reporter, mCherry-EGFP-LC3B (deposited by Jayanta Debnath) [44], and mitophagy reporter, mt-Keima (deposited by Michael Davidson) [14] and COX8-EGFP-mCherry (deposited by David Chan) [15], were purchased from Addgene. For A549 cybrid cells, the cells were seeded at the density of 4 × 10^4^/well in glass-bottom 24-well plates two days before transfection. According to the manufacturer’s protocol, Lipofectamine 3000 (Invitrogen, L3000001) was used for transfection. Specifically, we used 1 µg of DNA, and the ratio of P3000 to the 3000 reagent was 2:1. For fibroblasts, Human Dermal Fibroblasts Nucleofector Kit (Lonza, VPD-1001) was used for transfection according to the manufacturer’s protocol (2.5 µg DNA for 5 × 10^5^ cells). Experiments were conducted two days after transfection. Imaging was performed with the LSM 880 microscope using 63×/1.40 oil immersion objective lens at 37°C. The mCherry-EGFP-LC3B was excited at 488 and 561 nm separately and emitted fluorescence 500–580, and longer than 600 nm was collected. Images were acquired using Zen Black software (Carl Zeiss). Numbers and area of fluorescent particles for each channel were quantified using Fiji by thresholding images. For mt-Keima, following transfection, the cells were treated as indicated for 24 h and imaged via two sequential excitations (458 nm, green; 561 nm, red) using a 570–695 nm emission range. The laser power was set at the minimum output to allow the clear visualization of the mt-Keima signal and was separately adjusted for each experimental condition. At least 10 z-stacks with 0.45 µm thickness were acquired per sample in an experimental set. LysoTracker Green DND-26 (Invitrogen, L7526) was co-imaged using a 488 nm excitation and a 495–550 nm emission filter, where indicated. The ratio of the high F_543_:F_458_ ratio area to total mitochondrial area was used as an index of mitophagy. Ratio F_543_:F_458_ images were generated using the Ratio Plus plugin in Fiji. High F_543_:F_458_ ratio areas and total mitochondrial area were binarized, segmented and quantified in Fiji. Quantitative analysis of mitophagy in COX8-EGFP-mCherry-transfected A549 cybrid cells was also assessed using a flow cytometry-based approach. An arbitrary threshold of red fluorescence signal was used to indicate a mitophagy positive cell. Following transfection, cells were treated with either DMSO or different drugs as indicated. After 24 h of treatment, cells were trypsinized, washed once with DPBS and then resuspended into 1 ml of DPBS prior to analysis using a BD Accuri C6 flow cytometer. Cells were excited with a 488 nm laser, with emission assessed simultaneously using a 533/30 nm (FL1 detector) and a 670 LP filter (FL3 detector). Cells with a high FL3:FL1 ratio were selected, and their proportion was calculated.

#### Measurements of lysosomal proteolytic capacity by DQ Red BSA assay

Lysosomal proteolytic activity in cells was measured using DQ Red BSA (Life Technologies, D12051) – bovine serum albumin labeled with a red fluorophore. As a polymer, the signal of the fluorophore is quenched. When DQ-BSA is taken up by endocytosis, delivered to the lysosomes and degraded by the lysosomal proteases, it releases monomers that emit fluorescence. Cells were plated at a density of 2 × 10^4^ cells/well in a glass-bottom 96-well plate two days before experiments. The medium was replaced with DMEM high glucose containing 10 μg/ml DQ-Red-BSA and incubated for 1 h at 37°C. After the incubation, the plate was washed twice with DPBS, and the medium was replaced with 100 μl of the recording medium, which was phenol red-free DMEM (Gibco, A1443001) with 10 mM glucose, 1 mM glutamine, 10 mM HEPES, adjusted to pH 7.4. Lysosomal protease activity was measured as fluorescence in the CLARIOstar microplate reader (excitation/emission = 590/620 nm) every 5 min for 4 h. The slope of the linear range was used for analysis to determine the rate of DQ-BSA hydrolysis, which is a function of lysosomal proteases [45].

#### Assessment of lysosomal number and size

To assess lysosomal functions, cells were seeded (3 × 10^4^/well for A549 cybrid cells; 1 × 10^4^/well for fibroblasts) in glass-bottom 24-well plates, 2 days before imaging. Cells were washed twice with DPBS and then incubated with 200 nM LysoTracker Green DND-26 (Invitrogen, L7526) for 20 min at 37°C in the recording medium [45]. The cells were imaged with an LSM 880 Airyscan (Carl Zeiss) confocal microscope using Fluar 63×/1.40 oil immersion objective lens at 37°C. LysoTracker Green DND-26 was excited at 488 nm and collected fluorescence longer than 510 nm. Image analysis was performed using Fiji by thresholding and binarizing to evaluate the number of lysosomes and their areas for each imaged cell.

#### The mitochondrial oxygen consumption rate

Cellular aerobic respiration was measured using the Seahorse Bioscience XFe-96 bioanalyzer. Two days before the experiment, A549 cybrid cells (1 × 10^4^ cells/well) were seeded on XF96 cell culture microplates (Agilent 102,416-100). On the day of the experiment, the culture medium was replaced with Seahorse XF Base medium (Agilent 103334-100) supplemented with 1 mM pyruvate (Gibco 11360070), 2 mM glutamine (Gibco 25,030,081) and 10 mM glucose (Gibco, A2494001) and incubated for 30 min at 37°C in a CO_2_-free incubator before putting into the Seahorse Analyzer. After measuring basal respiration, cellular respiration was then measured under the conditions with chemicals added in the sequential order: oligomycin (5 µM), FCCP (1 µM, 2 µM), and rotenone+antimycin A (0.5 µM each). After the assay, the numbers of cell nuclei (cell numbers) in each well that stained with Hoechst 33342 (5 µM for 30 min; Thermo Scientific 62249) and counted by ImageXpress were used for the normalization of the experiments.

### Mitochondrial membrane potential (ΔΨ_m_)

Cells were seeded 2–3 days prior to imaging (3 × 10^4^/well for A549 cybrid cells; 1 × 10^4^/well for fibroblasts) in glass-bottom 24-well plates. Cells were washed twice with the recording medium and then stained with 25 nM tetramethylrhodamine methyl ester (TMRM; Sigma-Aldrich, T5428) for 30 min at 37°C. Cells were imaged with an LSM 880 (Carl Zeiss) confocal microscope using a Fluar 63×/1.40 oil immersion objective lens at 37°C. TMRM was excited with a 561 nm Argon laser with an output power of 0.2 mW. MBS 488/561 was used as a beam splitter and emitted fluorescence collected at 564–740 nm. Images were acquired using Zen Black software (Carl Zeiss), and fluorescence intensity was quantified using Fiji with the same threshold across all samples. To quantitatively describe mitochondrial morphology, mitochondrial network fragmentation was applied by Rehman et al [46]. Briefly, thresholding images obtained from previous steps using Fiji were further binarized, and the mitochondrial structure of each cell was counted using a particle counting plugin (>0,1 μm^2^) and normalized to the total mitochondrial area (μm^2^) per cell to obtain the mitochondrial fragmentation count (MFC). To be specific, MFC = (number of particles x 100)/total mitochondrial area.

### Transmission electron microscopy (TEM) imaging

Cells cultured on coverslips were fixed in EM fixative (2% glutaraldehyde + 2% paraformaldehyde in 0.1 M sodium cacodylate) for 1 h, followed by washings with 0.1 M Cacodylate Buffer. They were then fixed in 1% osmium tetroxide and 1% potassium ferricyanide in 0.1 M sodium cacodylate, followed by sequential ethanol dehydration. The coverslips were then embedded in Epoxy Resin (Araldite) Kit (Agar Scientific Ltd., CY212) according to the standard protocol. Embedded coverslips were sectioned to 50 nm using an ultra-microtome with a diamond knife and mounted onto TEM-compatible copper grids. The grids were then stained with lead citrate for 3 min before proceeding for Imaging. Images were acquired using the Jeol 1400 Transmission Electron Microscope. Images were taken at a magnification between 800X and 1200X (digital magnification). Autophagosomes were segmented using DeepMIB software [47]. Images were imported in Fiji to quantify autophagosomal area and number. Autophagosomal structures were manually traced to determine the area. To quantify autophagosome numbers per square micron, a 16 × 16 μm grid was overlaid on each image to quantify structures, including 90% of cytoplasmic area, and the numbers were presented per μm^2^ of cytoplasm.

#### Quantification and statistical analysis

All statistical analyses, unless otherwise stated in figure legends, were carried out using GraphPad Prism 8. To compare means between two groups, a two-tailed unpaired t-test was used for normally distributed data. One/two-way ANOVA with Tukey’s multiple comparisons test was performed for multi-group (at least three) comparisons. Data are presented as graphs displaying mean ± S.D., of at least three independent biological replicates. Means of control samples on immunoblotting or immunofluorescence are typically centered at one (or 100%) to ensure easier comparisons unless otherwise noted. Differences were only considered to be statistically significant when the *p* value was less than 0.05. Estimated *p* values are either stated as the actual values or denoted by *p < 0.05, **p < 0.01, ***p < 0.001, ****p < 0.0001. No statistical method was used to predetermine sample size, and replicates are shown in Figure legends. The investigators were not blinded to allocation during experiments and outcome assessment.

## Supplementary Material

A3243G mitophagy manuscript Suppl info_Final.docx
