# Supplementary material for: Inhibition of the PI3K-AKT-MTORC1 axis reduces the burden of the m.3243A>G mtDNA mutation by promoting mitophagy and improving mitochondrial function
Source: Autophagy. 2024 Dec 12;21(4):881–96. doi: 10.1080/15548627.2024.2437908 (PMC11925111; doi:10.1080/15548627.2024.2437908)
Supplement: A3243G mitophagy manuscript Suppl info_Final.docx [file KAUP_A_2437908_SM3290.docx]

**Inhibition of the PI3K-AKT-MTORC1 axis reduces the burden of the m.3243A>G mtDNA mutation by promoting mitophagy and improving mitochondrial function**

Chih-Yao Chung^1,#^, Kritarth Singh^1,#^, Preethi Sheshadri^1^, Gabriel E Valdebenito^1^, Anitta R Chacko^1^, María Alicia Costa Besada^1,2^, Xiao Fei Liang^1^, Lida Kabir^1^, Robert D S Pitceathly^3,4^, Gyorgy Szabadkai^1,5,6^, Michael R Duchen^1,*^

1 Department of Cell and Developmental Biology and Consortium for Mitochondrial Research, UCL, Gower Street, London WC1E 6BT, UK

2 Cellular and Molecular Neurobiology of Parkinson's Disease, Research Center for Molecular Medicine and Chronic Diseases (CIMUS), University of Santiago de Compostela, Spain

3 Department of Neuromuscular Diseases, UCL Queen Square Institute of Neurology, London WC1N 3BG, UK

4 NHS Highly Specialised Service for Rare Mitochondrial Disorders, Queen Square Centre for Neuromuscular Diseases, The National Hospital for Neurology and Neurosurgery, London WC1N 3BG, UK

5 Department of Biomedical Sciences, University of Padua, via G. Colombo 3, 35100, Padua, Italy

6 The Francis Crick Institute, 1 Midland Rd, London NW1 1AT, UK

# Equal contribution

*Correspondence: m.duchen@ucl.ac.uk

**SUPPLEMENTARY INFORMATION**

Supplementary Materials and Methods

Supplementary Tables 1-2

Supplementary Figures 1-5

Supplementary References

**Supplementary Materials and Methods**

***Quantification of relative mtDNA copy number***

Quantitative PCR with primers for the mtDNA tRNA^Leu(UUR)^ and with primers for the nuclear B2M (beta-2-microglobulin) were used to determine the relative mtDNA copy number of cells [1]. PCR mix consists of 2 μl of template DNA (3 ng/μl), 2 μl of primer pair (final concentration of 400 nM), 12.5 μl of SYBR Green JumpStart Taq ReadyMix (Sigma-Aldrich, S4438) and 8.5 μl of DNase/RNase-free H_2_O. The thermal cycling conditions were as follows: 50°C for 2 min, 95°C for 10 min and then 40 cycles of 95°C for 15 s and 62°C for 1 min. Each sample has three technical replicates. The following equation was used to determine the relative mitochondrial DNA content, 2 x 2^ΔCT^, where ΔC_T_ is nuclear DNA CT value subtracted by mtDNA CT value. All primer pairs used can be found in Table S2.

***Quantitative reverse transcriptional PCR (RT-qPCR)***

RNeasy Mini Kit (Qiagen, 74104) was used to extract RNA. The concentrations and quality of RNA samples were quantified using NanoDrop. SuperScript IV First-Strand Synthesis System Kit (Invitrogen, 18091050) was used for cDNA synthesis from 1 μg total RNA according to manufacturer instructions. PCR mix consists of 2 μl of cDNA, 2 μl of primer pair (final concentration of 400 nM), 12.5 μl of SYBR Green JumpStart Taq ReadyMix and 8.5 μl of DNase/RNase free H_2_O. The thermal cycling conditions were as follows: 50°C for 2 min, 95°C for 10 min and then 40 cycles of 95°C for 15 s and 62°C for 1 min. Quantification was made using the comparative threshold method (2^-ΔΔCT^), normalizing data to *TUBB* and untreated samples [2]. All primer pairs used can be found in Table S2.

***Immunofluorescence***

For A549 cybrid cells, the cells were seeded at 1 x 10^5^ cells per well in 6-well plates on 22 mm glass coverslips. On the experiment day, cells were replenished with regular media for 1 h to prevent starvation-induced autophagy. After 1 h, treatment conditions resumed with or without 50 μM chloroquine for 6 h. The cells were then washed with PBS twice and fixed in 4% paraformaldehyde for 20 min at room temperature and permeabilized with 0.2% Triton X-100 (Sigma-Aldrich, T9284) in PBS for 5 min. Following washes with 0.2% Triton X-100 PBS, coverslips were incubated with primary anti-LC3B for autophagosomes/lysosomes (1:500) and anti-CYCS for mitochondria (1:200; BD Pharmingen, 556432) antibodies in 3% BSA (Sigma-Aldrich, A9418), 0.2% Triton X-100 PBS at 4°C overnight. The following day, cells were washed with 0.2% Triton X-100 PBS and incubated with Alexa Fluor 488-conjugated (goat anti-rabbit, 1:200; Invitrogen, A11008) and Alexa Fluor 647-conjugated (donkey anti-mouse, 1:500; Invitrogen, A31571) secondary antibodies for visualization of the lysosomes and mitochondria, respectively for 1 h. Following three PBS washes, the nucleus was counterstained using 5 μg/mL Hoechst 33342 solution diluted 1:2000 in 0.2% Triton X-100 PBS for 5 min. The coverslips were washed one final time with 0.2% Triton X-100 PBS and mounted on a glass slide using Fluoromount-G mounting medium (SouthernBiotech, 0100-01) and stored at 4°C until imaging. Imaging was performed with the LSM 880 microscope using a 63x/1.40 oil immersion objective lens. Hoescht 33342 was excited at 405 nm for tricolor imaging, Alexa Fluor 488 at 488 nm, and Alexa Fluor 647 at 633 nm. Emitted fluorescence was collected at 410-480 nm, 500-650 nm and longer than 660 nm. Images were acquired using Zen Black software (Carl Zeiss). Image acquisition parameters settings were maintained for all samples within the experimental set. The colocalization coefficient (Pearson's R-value) of 647 nm and 488 nm emission signals, LC3B and CYCS, was quantified using Coloc 2 in Fiji.

**Table S1.** KEGG pathway enrichment of DEGS in dataset II using the GAGE method

| Direction | adj. Pval | nGenes | Pathways |
| --- | --- | --- | --- |
| Downregulated | 6.8e-08 | 34 | Herpes simplex virus 1 infection |
| Upregulated | 1.3e-05 | 16 | Focal adhesion |
|  | 8.0e-04 | 18 | **PI3K-AKT signaling pathway** |
|  | 1.3e-03 | 9 | AGE- AGER/RAGE signaling pathway in diabetic complications |
|  | 2.2e-03 | 13 | RAS signaling pathway |
|  | 2.2e-03 | 10 | Fluid sheer stress and atherosclerosis |
|  | 3.2e-03 | 8 | Hematopoietic cell lineage |
|  | 3.7e-03 | 8 | HIF-1 signaling pathway |
|  | 4.1e-03 | 14 | MAPK signaling pathway |
|  | 4.5e-03 | 6 | VEGF signaling pathway |
|  | 4.5e-03 | 7 | ECM-receptor interaction |
|  | 4.5e-03 | 8 | TNF signaling pathway |
|  | 7.5e-03 | 7 | Small cell lung cancer |
|  | 9.0e-03 | 7 | Amoebiasis |
|  | 9.8e-03 | 8 | **Autophagy** |

**Table S2**. Primer pairs used in this study.

| Target | Forward | Reverse | Source |
| --- | --- | --- | --- |
| m.3243A | CAGGGTTTGTTAAGATGGCAtA | TGGCCATGGGTATGTTGTTA | [3] |
| m.3243G | CAGGGTTTGTTAAGATGGCAtG |  |  |
| mitochondria | CACCCAAGAACAGGGTTTGT | TGGCCATGGGTATGTTGTTA | [1] |
| nucleus | TGCTGTCTCCATGTTTGATGTATCT | TCTCTGCTCCCCACCTCTAAGT |  |
| TUBB | ATGGACGAGATGGAGTTC | TTGAGTAAGACGGCTAAGG | [2] |
| PPARGC1A | TGATGACAGCGAAGATGA | AGAAGAACAAGAAGGAGACA |  |
| PPARGC1B | ACACTGACTACGATTCCAA | TCTGAGGTATTGAGGTATTCC |  |
| NDUFV2 | ACTCCTGAGAATAACCCTGATACT | GCAACCTTGTTCATAGCAGAGA |  |
| SDHA | ATGGAAGGTCTCTGCGATATGAT | TGCTCTTATGCGATGGATGGA |  |
| SDHD | TCACCGAGCCACCATTCT | CATCGCAGAGCAAGGATTCAA |  |
| CYCS | AAGATTGTGCCACTGCACTCAAGC | AGGTGAGCACAACAGGAACTGGAA |  |
| COX4I | CGGTGCCATGTTCTTCATCGGTTT | TCATGTCCAGCATCCTCTTGGTCT |  |
| COX5B | GGAAGACCCTAATTTAGTCCCCT | CCAGCTTGTAATGGGCTCCAC |  |


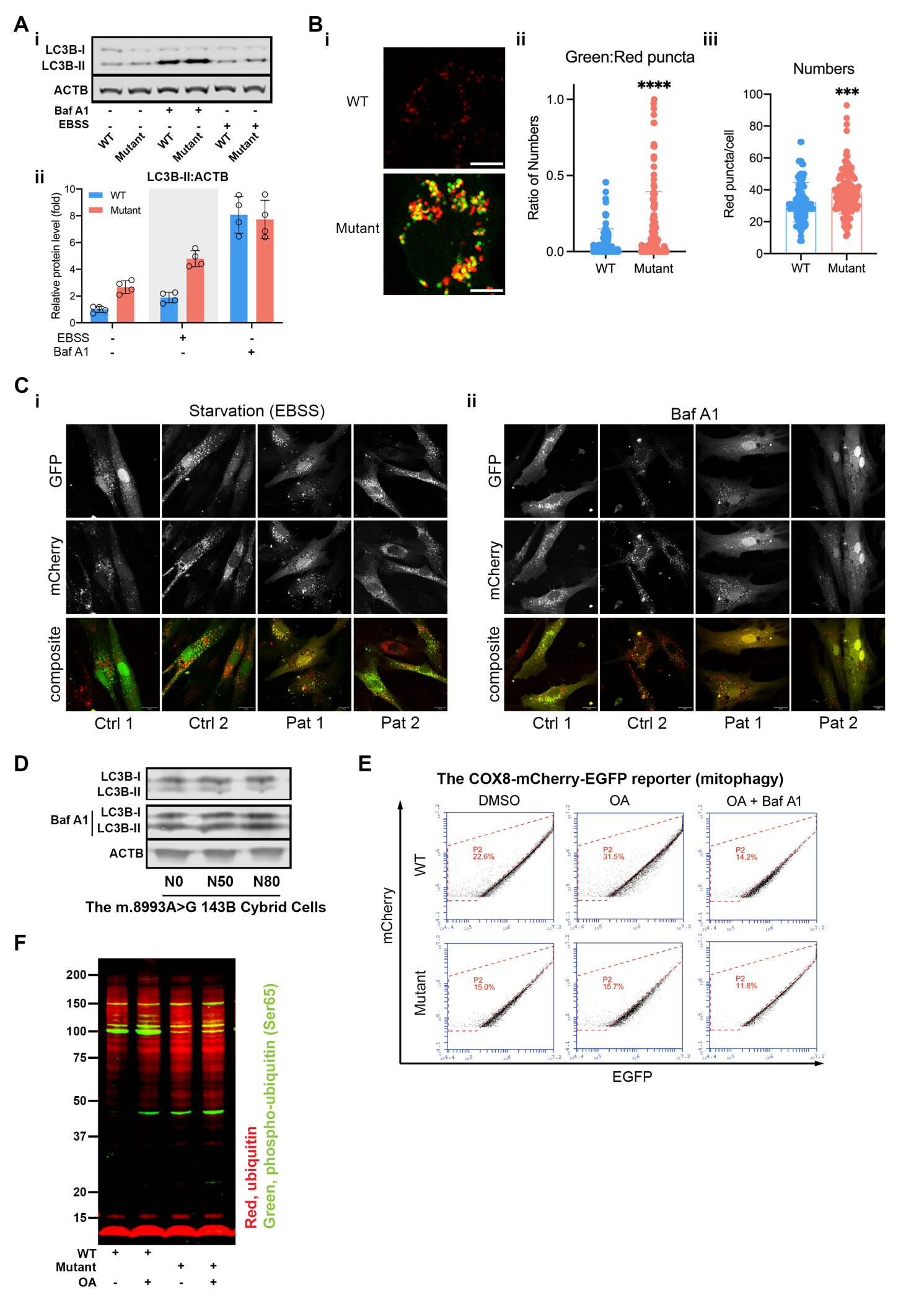


**Figure S1.** Other supporting results from patient fibroblasts and A549 cybrid cells for assessing lysosomes, autophagy and mitophagy. (**A**) Immunoblot of LC3B in the A549 cybrid cells under basal, starvation (EBSS) or bafilomycin A_1_ (Baf A1; 100 nM) conditions (n = 3-4) also shows an accumulation of LC3B-II in the mutant cells without an overall fold increase in autophagic flux (i.e., the conversion of LC3B-I to LC3B-II). (**B**) Live cell imaging of mCherry-GFP-LC3B in A549 cybrid cells (i). The ratio of green:red puncta (ii) and autophagosome numbers (iii) were further quantified, showing an increase of green:red puncta and total numbers in the mutant cells. The plot represents data from n > 100 cells from independent experiments. Scale bar: 10 μm. (**C**) Representative images of control and patient fibroblasts transfected with the mCherry-GFP-LC3B showing the relative numbers of mCherry and GFP+mCherry puncta in EBSS (i) and the Baf A1 (ii) condition. Scale bar: 20 μm. (**D**) Immunoblotting of LC3B (± Baf A1) in 143B cybrid cells bearing the m.8993T>G mutation showed no difference in autophagic flux among cells with 0% (N0), 50% (N50) and 80% (N80) mutant loads (n = 3 independent experiments). (**E**) Quantification of mitophagy in COX8-EGFP-mCherry transfected A549 cybrid cells under normal (DMSO), oligomycin+antimycin A (OA, mitophagy stimulated) OA+Baf A1 (mitophagy inhibited condition) using flow cytometry. P2 represents the percentage ‘mitophagic population’. (**F**) Immunoblot of total ubiquitin and phospho-ubiquitin (Ser65) in OA-treated A549 cybrid cells to assess PINK1 activation. The blot is representative of three independent experiments. Data in (**A**-**B**) are represented as mean ± S.D. and were analyzed by unpaired t test for cybrid cells (* *p* < 0.05, ** *p* < 0.01, *** *p* < 0.001, **** *p* < 0.0001).


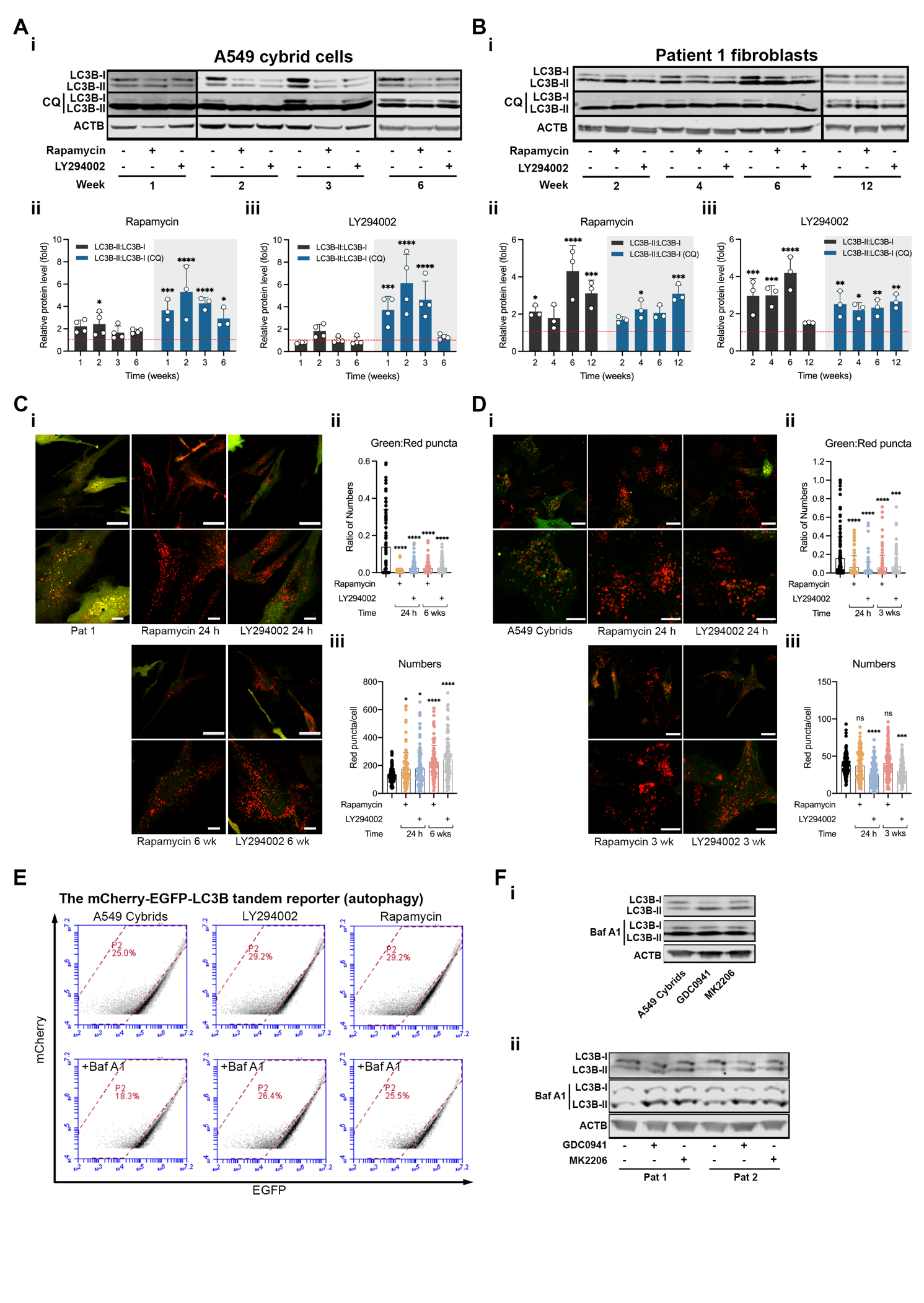


**Figure S2.** Autophagy flux is increased upon inhibition of the PI3K-AKT-MTORC1 axis in the m.3243A>G mutant cells. (**A**) In A549 cybrid cells, immunoblotting of LC3B with or without CQ (50 μM for 5 h on the day of experiments) over 6-week drug treatments (i) showed a significant increase in autophagic flux in drug-treated cells (ii and iii; n = 3 independent experiments). (**B**) In patient 1 fibroblasts, immunoblotting (i) of LC3B with or without CQ (50 μM for 5 h on the day of experiments) over 12-week drug treatments (ii and iii) showed a significant increase in autophagic flux in drug-treated cells (n = 3-4 of independent experiments). (**C**) Representative images of Pat 1 fibroblasts, transfected with the autophagy reporter, mCherry-GFP-LC3B and treated with LY or RP for the indicated time points (i; scale bar: 50 μm for full-scale images and 10 μm for zoomed-in images). The ratio of green:red puncta (ii) and only red puncta (iii), which indicates the maturation of autophagosomes to autolysosomes, were further quantified in response to different treatment durations. The plots represent data from n > 100 cells from five independent experiments. (**D**) Confocal imaging of mCherry-GFP-LC3B in A549 cybrid cells treated with LY or RP for the indicated time points (i; scale bar: 20 μm for full-scale images and 10 μm for zoomed-in images). The ratio of green:red puncta (ii) and only red puncta (iii), which indicates the maturation of autophagosomes to autolysosomes, were further quantified in response to different treatment durations. The plots represent data from n > 100 cells from three independent experiments. (**E**) Measurement of autophagy flux by flow cytometry in mCherry-GFP-LC3B transfected A549 cybrid cells treated with LY or RP either alone or in combination with Baf A1, showing an increase of cells with low GFP intensity (P2 gate) when treated with RP and LY, while Baf A1 treatment reversed the effect. (**F**) Immunoblotting of LC3B (± Baf A1) in A549 cybrid cells (i) and in patient fibroblasts (ii) treated with GDC or MK over 6 weeks demonstrated the upregulated autophagic flux in the drug-treated cells. Blots are representative of three independent experiments. Data are represented as mean ± S.D. and were analyzed by one-way ANOVA with Tukey’s multiple comparisons test (* *p* < 0.05, ** *p* < 0.01, *** *p* < 0.001, **** *p* < 0.0001).


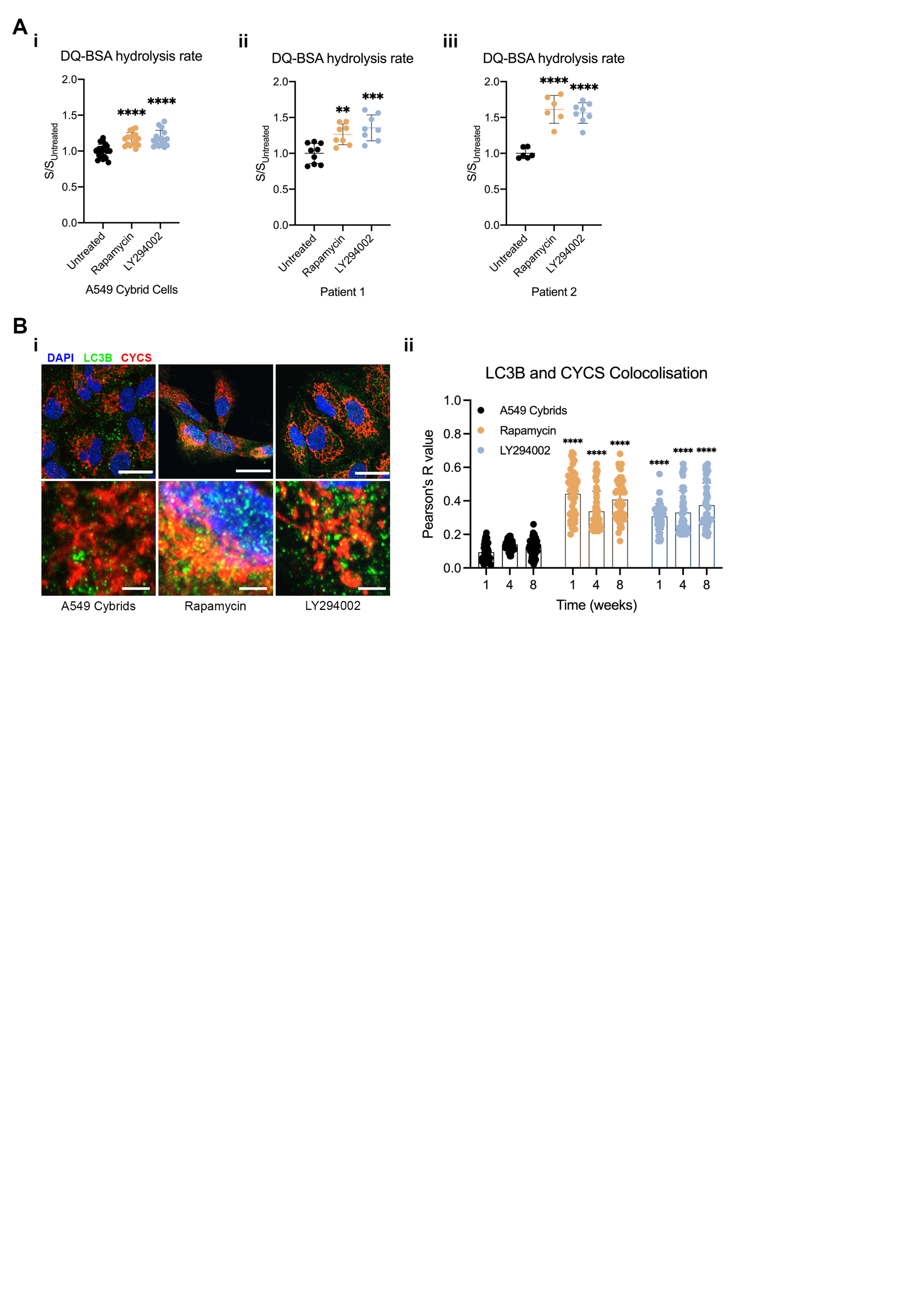


**Figure S3.** Lysosomal proteolytic capacity and mitophagy are improved following the inhibition of the PI3K-AKT-MTORC1 axis. (**A**) Lysosomal proteolytic activity measured by DQ-BSA assay showed that the activity in the mutant cells, both the A549 cybrid cells and patient fibroblasts, was partially recovered following inhibition of the PI3K-AKT-MTORC1 axis (n = 3 of independent experiments). (**B**) Representative images of LC3B and CYCS immunostaining in A549 cybrid cells treated with either RP or LY, indicating LC3B recruitment and degradation of mitochondria by mitophagy. Scale bar: 20 μm for full-scale images and 2 μm for zoomed-in images. Quantification of co-localization coefficient, Pearson’s R, between LC3B and CYCS signal intensity under untreated and drug-treated conditions at different time points. The plot represents data from n > 40 cells from three independent experiments. Data are represented as mean ± S.D. and were analyzed by one-way ANOVA with Tukey’s multiple comparisons test (* *p* < 0.05, ** *p* < 0.01, *** *p* < 0.001, **** *p* < 0.0001).


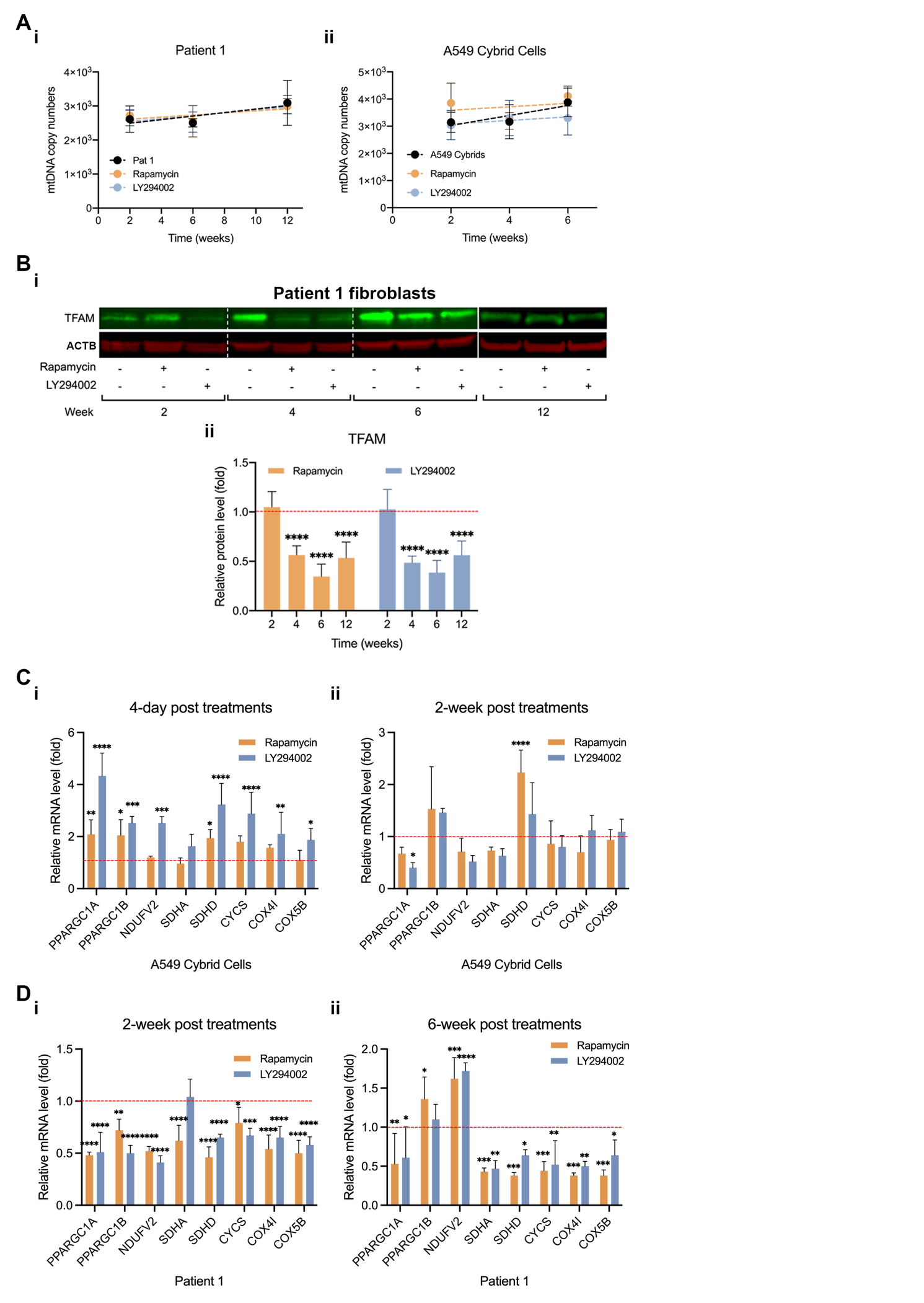


**Figure S4.** Mitochondrial biogenesis and changes in mtDNA copy number do not explain the reduction of mutant mtDNA burden. (**A**) Quantitative PCR assessing mtDNA copy numbers of patient 1 fibroblasts (i) and A549 cybrid cells (ii) over the 6- or 12-weeks of treatments (n = 3 independent experiments). (**B**) Immunoblot of TFAM in Pat 1 fibroblasts throughout 12-week drug treatment with RP and LY. (i). Quantification of relative TFAM levels under RP- and LY-treated conditions at different time points (n = 4). (**C**-**D**) RT-qPCR of *PPARGC1A-* and *PPARGC1B-*related genes in A549 cybrid cells (**C**) and in Pat 1 fibroblasts (**D**) at indicated time points showed a transient activation of PPARGC1A and PPARGC1B as well as significant changes in multiple mitochondrial-related proteins (n = 3). Data are represented as mean ± S.D. and were analyzed by one-way ANOVA with Tukey’s multiple comparisons test (* *p* < 0.05, ** *p* < 0.01, *** *p* < 0.001, **** *p* < 0.0001).


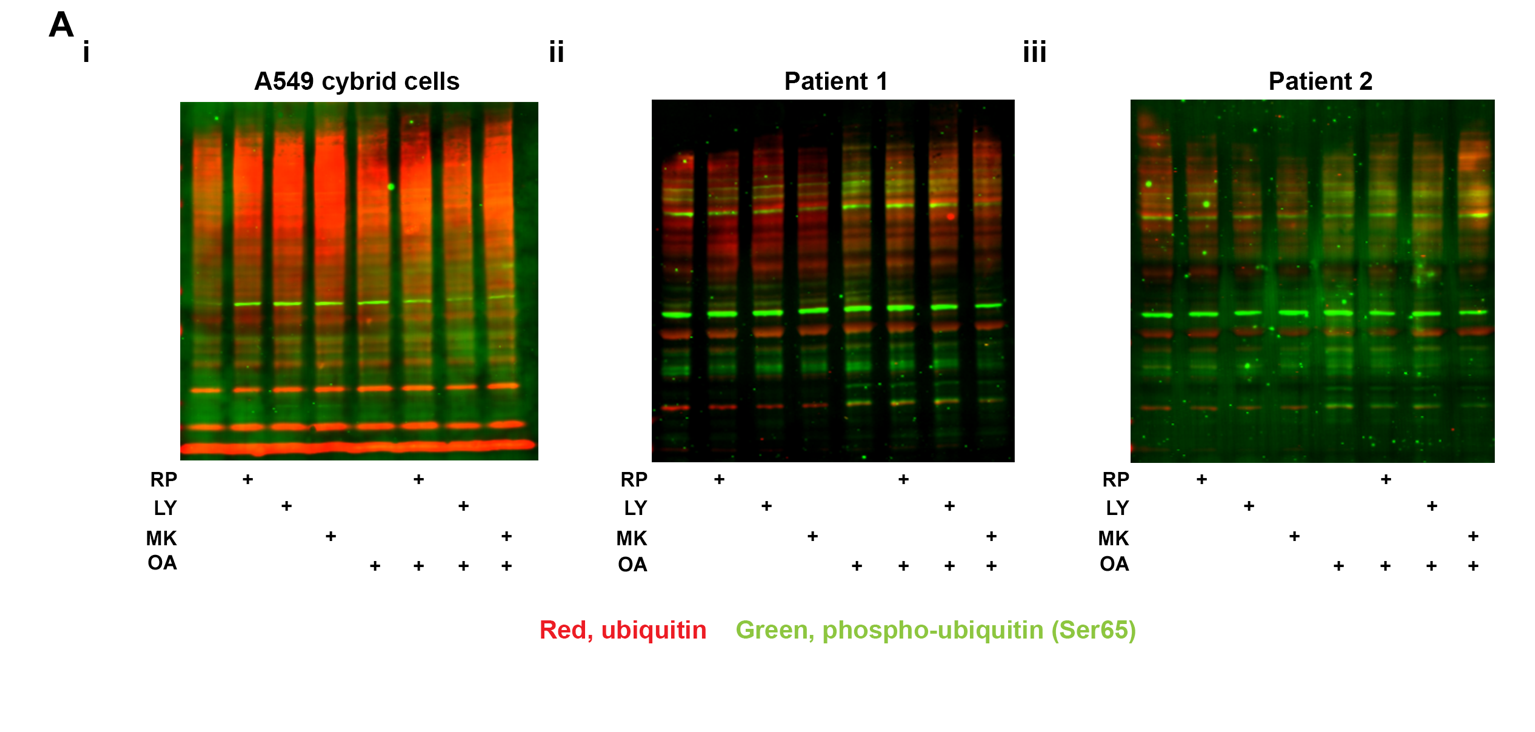


**Figure S5.** PINK1 activation is not affected in the m.3243A>G mutant cells following inhibition of the PI3K-AKT-MTORC1 axis. (**A**) Immunoblotting of total ubiquitin and phospho-ubiquitin (Ser65) in the PI3K-AKT-MTORC1 inhibited A549 cybrid cells (i), the Pat 1 fibroblasts (ii) and Pat 2 fibroblasts (iii), co-treated with oligomycin+antimycin A (OA) showed that PINK1 activity did not significantly improve after drug treatments. The blots are representative of three independent experiments.

**Supplementary References**

[1] Rooney JP, Ryde IT, Sanders LH, et al. PCR based determination of mitochondrial DNA copy number in multiple species. Methods Mol Biol. 2015;1241:23–38.

[2] Yao Z, Jones AWE, Fassone E, et al. PGC-1β mediates adaptive chemoresistance associated with mitochondrial DNA mutations. Oncogene. 2013;32(20):2592–2600.

[3] Wang J, Venegas V, Li F, et al. Analysis of mitochondrial DNA point mutation heteroplasmy by ARMS quantitative PCR. Curr Protoc Hum Genet. 2011;Chapter 19:Unit 19 6.
